# The molecular taxonomy of three endemic Central Asian species of *Ranunculus*(Ranunculaceae)

**DOI:** 10.1371/journal.pone.0240121

**Published:** 2020-10-05

**Authors:** Shyryn Almerekova, Natalia Shchegoleva, Saule Abugalieva, Yerlan Turuspekov

**Affiliations:** 1 Molecular Genetics Laboratory, Institute of Plant Biology and Biotechnology, Almaty, Kazakhstan; 2 Department of Botany, Institute of Biology, Tomsk State University, Tomsk, Russia; 3 Department of Biodiversity and Bioresources, Al-Farabi Kazakh National University, Almaty, Kazakhstan; 4 Agrobiology Faculty, Kazakh National Agrarian University, Almaty, Kazakhstan; University of Helsinki, FINLAND

## Abstract

Worldwide, the genus *Ranunculus* includes approximately 600 species and is highly genetically diverse. Recent taxonomic reports suggest that the genus has a monophyletic origin, divided into two subgenera, and consists of 17 sections. The Central Asian country of Kazakhstan has 62 species of the genus that have primarily been collected in the central part of the country. The latest collection trips in southern parts of the country have led to the description of a wider distribution area for *Ranunculus* and the identification of a new species *Ranunculus talassicus* Schegol. et A.L. Ebel from Western Tien Shan. Therefore, in this study, attempts were made to assess the molecular taxonomic positions of *R*. *talassicus* and two other species endemic to the Central Asian region *R*. *karkaralensis* Schegol. and *R*. *pskemensis* V.N. Pavlov in relation to other species of the genus, using internal transcribed spacer (ITS) molecular genetic markers. The ITS-aligned sequences of 22 local Central Asian accessions and 43 accession sequences available in the National Center for Biotechnology Information (NCBI) database allowed the construction of a maximum parsimony phylogenetic tree and a Neighbor-Net network. The results indicated that *R*. *talassicus* and *R*. *pskemensis* could be assigned to section *Ranunculastrum*. Additionally, an assessment of the network suggested that *R*. *pskemensis* was the rooting taxon for the group of species containing *R*. *talassicus*, and that *R*. *illyricus* L. and *R*. *pedatus* Waldst. & Kit. were founders of a prime rooting node for the *Ranunculastrum* section of the genus. The ITS-aligned sequences showed that *R*. *karkaralensis* was indifferent with respect to three other species in the *Ranunculus* section of the genus, i.e., *R*. *acris* L., *R*. *grandifolius* C.A. Mey., and *R*. *subborealis* Tzvelev. The study indicated that the assessments of ITS-based phylogenetic tree and Neighbor-Net network provided new insights into the taxonomic positions of three endemic species from Central Asia.

## Introduction

The Ranunculaceae Juss., or buttercup family, has a worldwide distribution and comprises more than 2500 species belonging to 59 genera. The family members span a wide range of different ecological conditions, especially in the Northern Hemisphere. The largest cosmopolitan genus of the family is *Ranunculus* L., which contains approximately 600 species distributed worldwide [1, 2]. Due to its wide distribution, this genus is highly genetically diverse. Consequently, classification of the genus *Ranunculus* is very complex and has not been fully completed. Initial worldwide classifications of *Ranunculus* were based on descriptions of achenes, flowers, roots [3], and fruit anatomy [4]. Later, Tamura revised the classification of the genus based on a reassessment of the achene structure [1, 2]. Tamura’s classification subdivided the genus into seven subgenera: *Pallasiantha*, *Coptidium*, *Ficaria*, *Batrachium*, *Crymodes*, *Gampsoceras*, and *Ranunculus*. In this classification, the subgenus *Ranunculus* was further subdivided into 20 sections [2]. Soon thereafter, DNA markers were implemented to clarify and discuss the phylogenetic relationships within Ranunculaceae [5–8]. Several publications using molecular markers have been dedicated to the *Ranunculus* genus and suggest that the molecular taxonomy of the genus is not congruent with the classifications based on botanical studies [8–10]. For instance, phylogenetic analyses of c. 200 species of *Ranunculus* based on sequences of the muclear ITS (nrITS) region [9] showed a high level of similarity to previous chloroplast DNA restriction site analyses [8] and differed from the previous classification based on phenetic studies.

DNA barcoding studies have often been used by different scientists for evaluating the generic delimitation and infrageneric classification of the genus *Ranunculus* [9–15]. Most of these studies have relied on the use of nucleotide sequences of the internal transcribed spacer (ITS) of the nuclear ribosomal DNA (rDNA) region, as well as plastid regions. ITS are widely used for differentiating between the generic and infrageneric levels of plant phylogenetics [16] and are one of the most powerful barcode DNA markers available [17]. ITS markers have been confirmed to be useful for clarifying the phylogenetic relationships between closely related species and genera in Ranunculaceae [18–21]. However, a complete study of the taxonomy of the genus was based on using both DNA markers and morphological data, with the division of 226 taxa into two subgenera and 17 sections [14].

The Central Asian country of Kazakhstan is the ninth largest country in the world and contains a rich array of flora, including a wide diversity of endemic and rare plant species. According to the Flora of Kazakhstan, the country has 62 species of *Ranunculus* [22], including the genus *Batrachium* (DC.) Gray. Previously, the botanical descriptions of Kazakh buttercups were related to the spatial distribution of the species within different floristic regions of Kazakhstan [23]. The botanical taxonomy of the genus *Ranunculus* has primarily been studied using samples collected in Central Kazakhstan [24, 25]. For instance, Schegoleva (2014) [24] presented the taxonomic composition of *Ranunculus* in Central Kazakhstan as consisting of 19 species, which were part of the subgenera *Ranunculus* and *Batrachium*. As a result of the collection trips in this region, a new *Ranunculus* species *R*. *karkaralensis* Schegoleva was claimed to have been identified [26]. The author noted that *R*. *karkaralensis* is similar to *R*. *grandifolius* C.A. Meyer and differs from it in terms of the form and proportions of the sheet plate of basal leaves and the absence of a well-developed horizontal rhizome [26].

Until recently, very little effort has been made to compile a botanical description of *Ranunculus* species in the southern part of the country, where a new species *R*. *talassicus* Schegol. et A.L. Ebel from Western Tien Shan was recently described [27]. It was suggested that *R*. *talassicus* is morphologically close to *R*. *alaiensis* Ostenf. from Pamir-Alai and differs from it in terms of having triple-dissected basal leaves [27]. As these newly identified species have not been analyzed using DNA markers, the purpose of this study was to clarify the molecular taxonomy of *R*. *karkaralensis* and *R*. *talassicus* based on ITS markers.

This work is part of a national program aiming to evaluate the genetic variation in the wild flora of Kazakhstan [28]. The project, along with DNA barcoding analyses of wild plant species [29–32], is also incorporating studies using simple sequence repeats (SSR) [33], amplified fragment length polymorphism (AFLP) [34], and single nucleotide polymorphism (SNP) [35] markers in a collaboration between local botanists and geneticists.

## Materials and methods

### Plant materials

**Fig 1:**
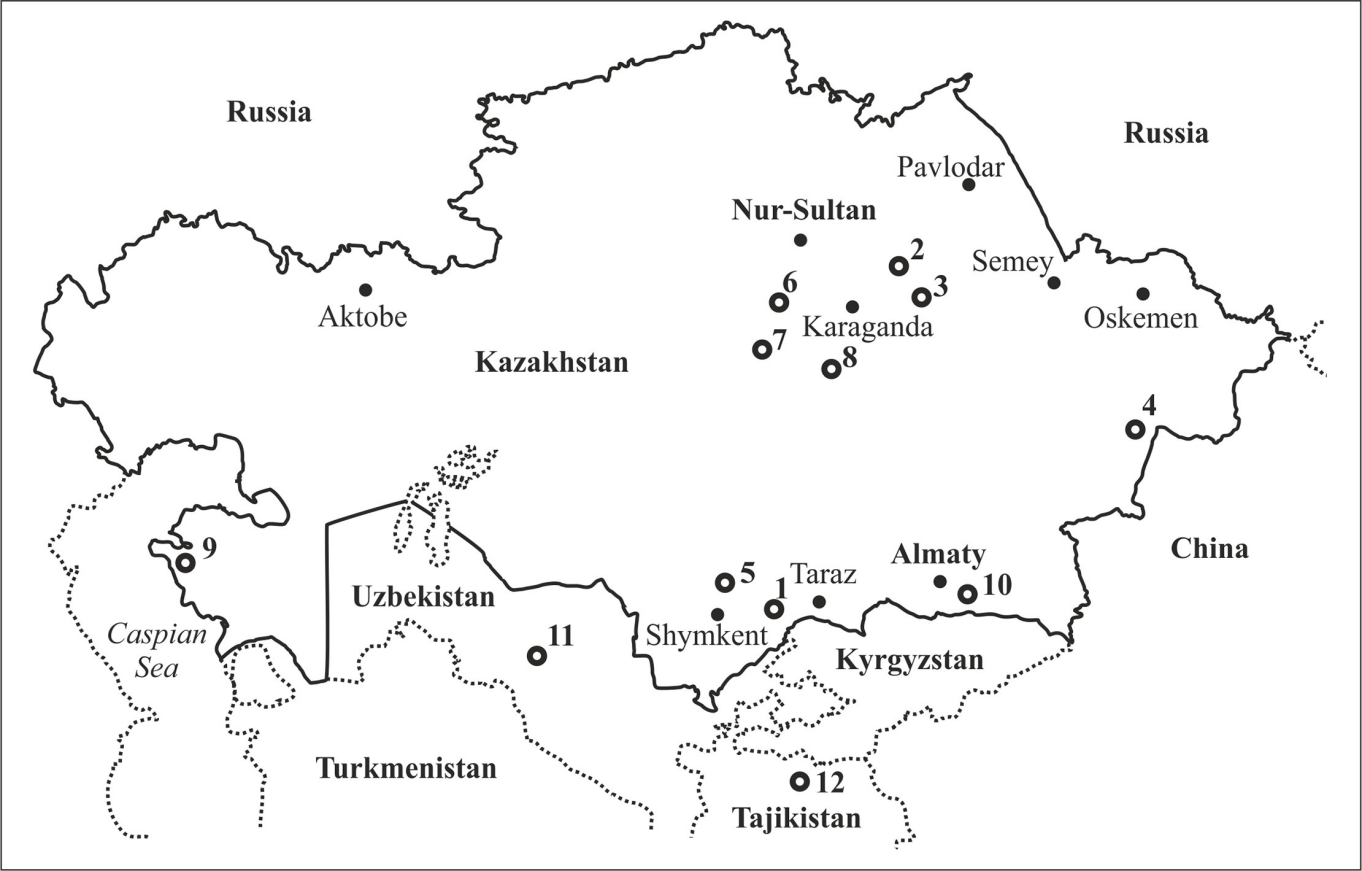
Collection sites of *Ranunculus* species in Kazakhstan, Uzbekistan, and Tajikistan. 1 *− R*. *rubrocalyx* Kom., *R*. *songaricus* Schrenk, *R*. *paucidentatus* Schrenk, *R*. *polyrhizos* Stephan ex Willd. (two populations), *R*. *talassicus* Schegol. et A.L. Ebel, *R*. *regelianus* Ovcz., *R*. *olgae* Regel, *R*. *laetus* Wall. ex Hook.f. & Thomson., *R*. *polyanthemos* L. (two populations), and *R*. *pskemensis* V.N. Pavlov; 2 − *R*. *polyanthemos*; 3 − *R*. *subborealis* Tzvelev and *R*. *grandifolius* C.A. Mey.; 4 − *R*. *natans* C.A. Mey.; 5 − *R*. *oxyspermus* Willd.; 6 − *R*. *repens* L.; 7 − *R*. *sceleratus* L. and *R*. *polyrhizos*; 8 − *R*. *pedatus*, *R*. *platyspermus* Fisch. (two populations), and *R*. *karkaralensis* Schegol.; 9 − *R*. *platyspermus*; 10 − *R*. *albertii* Regel & Schmalh.; 11 − *R*. *linearilobus* Bunge (Uzbekistan); 12 − *R*. *alaiensis* Ostenf. (Tajikistan).

Plant materials for genetic analyses were collected during the period 2015 to 2019 from different parts of Kazakhstan (Fig 1). The geographic locations of the collection sites are given in S1 Table. The permission for field work at the Sairam-Ugam National Nature Park (Turkestan region, Kazakhstan) was given by the authorities of the Park (S1 Appendix).

**Fig 2:**
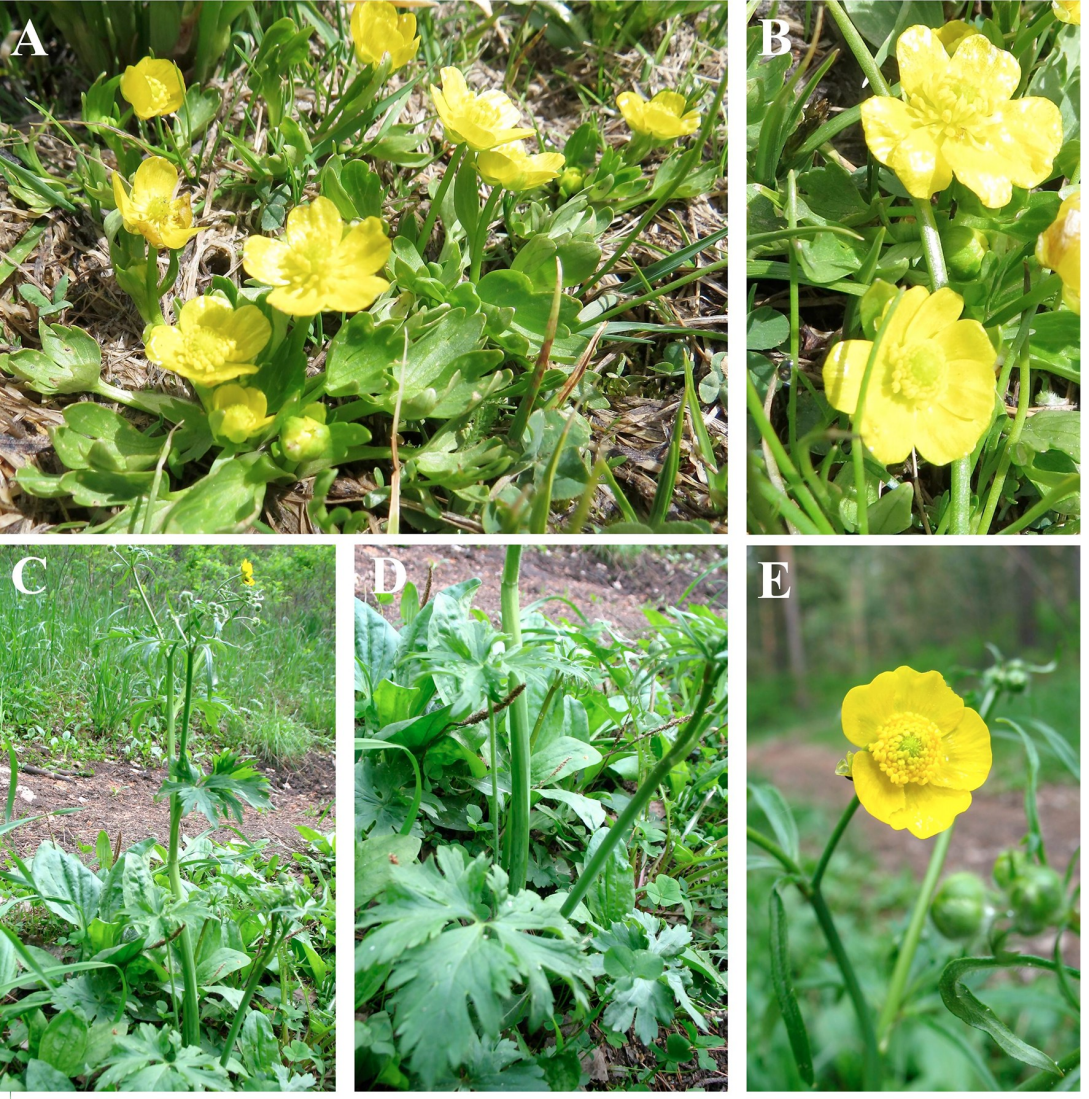
Habitus of *R*. *talassicus* (A and B) and *R*. *karkaralensis* (C, D, and E) collected in Kazakhstan.

Two endemic Kazakhstan species *R*. *talassicus* and *R*. *karkaralensis* (Fig 2), which were recently described as new species, were included in the phylogenetic analysis.

The study also included *R*. *pskemensis* V.N. Pavlov (species endemic to Uzbekistan), first sampled in Kazakhstan (Turkestan region) in 2019. The holotypes of the new species were deposited in TK (Herbarium at the Tomsk State University, Tomsk, Russian Federation), and isotypes were deposited in TK, AA (Herbarium at the Institute of Botany and Phytointroduction, Almaty, Kazakhstan), and TASH (Herbarium at the Institute of Botany, Tashkent, Uzbekistan) for *R*. *talassicus* and in TK for *R*. *karkaralensis*.

### DNA isolation, amplification, and sequencing

Total genomic DNA was isolated from dried leaf material of the species studied using the CTAB (cetyl trimethylammonium bromide) protocol [36]. The quality and concentration of DNA were assessed using a NanoDrop 2000 spectrophotometer (Thermo Fisher Scientific, USA). The internal transcribed spacers 1 and 2 (ITS) and the 5.8S rRNA gene were amplified using primers ITS1nF (5’-AGAAGTCGTAACAAGGTTTCCGTAGG-3’) and ITS4nR (5’-TCCTCCGCTTATTGATATGC- 3’) with an annealing temperature of 58°C [37]. Polymerase chain reaction (PCR) products were checked on 1.5% agarose gels. Single bands with the expected size of approximately 650 base pairs (bp) were cut out from the gel and subsequently purified using ULTRAPrep® Agarose Gel Extraction Mini-Prep Kits (AHN Biotechnologie GmbH, Nordhausen, Germany) according to the protocol provided by the company. Purified PCR products were amplified with forward and reverse primers separately using the BigDye Terminator v.3.1Cycle Sequencing Kit (Applied Biosystems, USA). The ethanol/EDTA method [38] was used for precipitation of the products. Further purified PCR products were sequenced in forward and reverse directions using the ABI 3130 DNA sequencer (Applied Biosystems, USA).

### Alignment, phylogenetic, and haplotype network analyses

Maximum Likelihood (ML) [39], Maximum Parsimony (MP) [40], and Neighbor-Joining (NJ) [41] methods were used for the phylogenetic analysis of *Ranunculus* species in MEGA software [42]. Bootstraps with 100, 500, 1000, 5000, and 10,000 replications were used for each method (S2 Appendix).

For reconstruction of the phylogenetic tree, 29 samples of 22 species collected in Kazakhstan, Uzbekistan, and Tajikistan (S1 Table) were sequenced, and 43 ITS sequences were downloaded from NCBI. The set of sequences consisted of 67 *Ranunculus* samples, including 22 local samples (one per species); 43 samples from NCBI; and 2 outgroup species, i.e., *Trollius altaicus* C.A. Mey. and *T*. *ledebourii* Rchb. New sequences were deposited in GenBank and are listed in S1 Table. In the phylogenetic analyses, the names of the sections and subgenera are given, according to Hörandl and Embadzade [14] and Schegoleva [43]. Genetic relationships between haplotypes were estimated using the Neighbor-Net algorithm in SplitsTree4 software [44]. The aligned sequences were converted into a Nexus file format in DNASP v5.10 [45] for analysis by SplitsTree software.

## Results

The length of the ITS sequences varied from 573 to 577 base pairs (bp) for 65 *Ranunculus* samples, without including the outgroup species, and up to 591 bp including the two outgroups. In general, out of the 591 aligned ITS nucleotides, 206 bp were polymorphic, including 121 bp in ITS1, 14 in 5.8S, and 71 in ITS2. The total variability of the ITS sequences was 34.85%, with 26 nucleotide sites appearing as singletons, and 159 sites registered as parsimonious informative sites.

**Fig 3:**
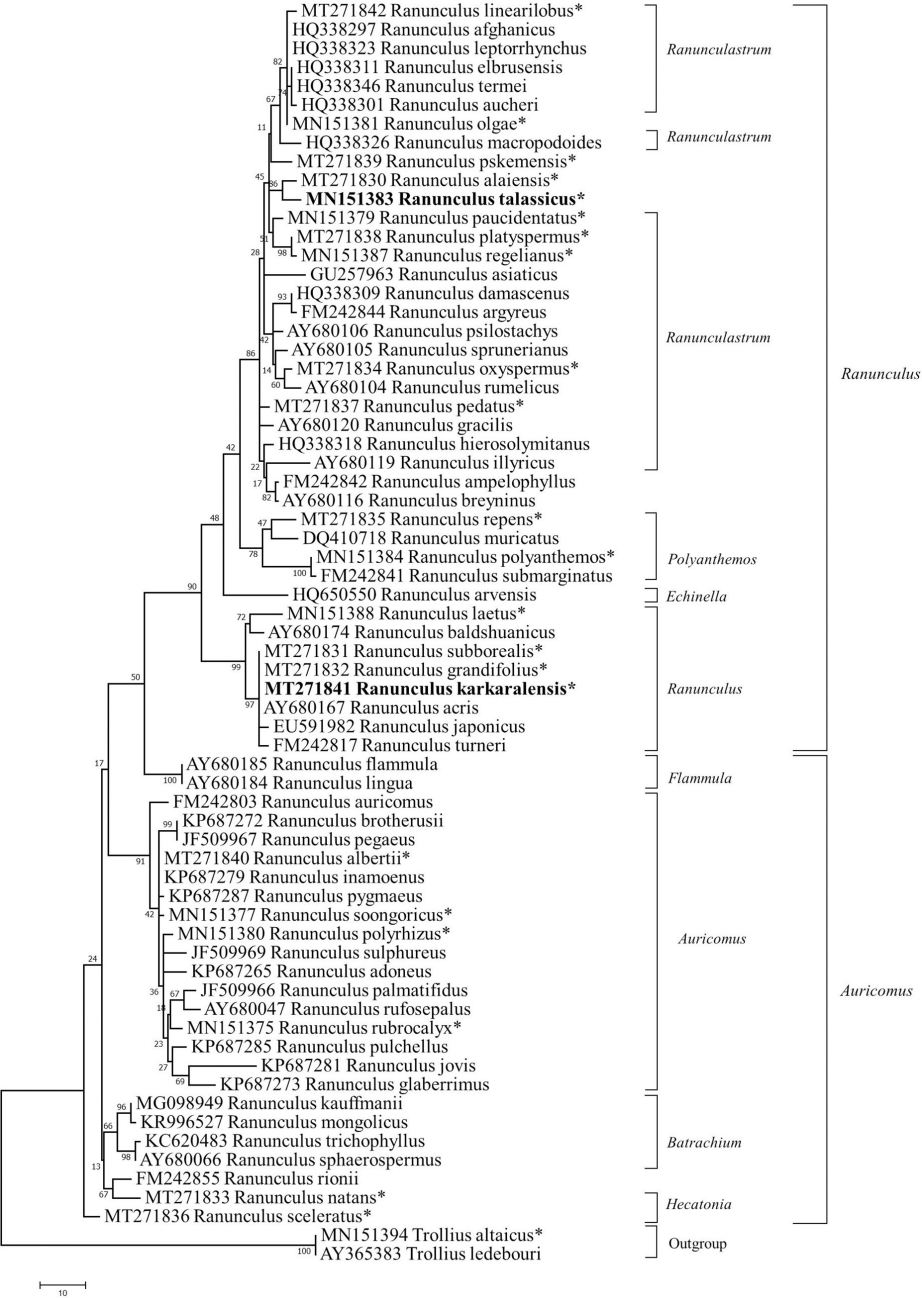
Maximum parsimony (MP) phylogenetic tree of *Ranunculus* species and two outgroup taxa constructed using internal transcribed spacer (ITS) sequences. The classification is given according to Hörandl and Emadzade, 2012. * denotes *Ranunculus* species analyzed in this study. The numbers above the branches represent MP bootstrap values. New species are highlighted in bold.

The alignment of ITS sequences for the 65 *Ranunculus* samples collected in Kazakhstan, neighbouring Central Asian countries, and Genbank was used for the construction of ML, MP, and NJ phylogenetic trees. An assessment of the resulting phylogenetic trees of the three methods suggested that the MP method provided clusters with the highest bootstrap values (Fig 3, S2 Appendix). Therefore, in the following steps of the study, the MP phylogenetic tree was selected for the analysis. The MP phylogenetic tree classified samples into two large *Ranunculus* and *Auricomus* subgenera clades, with further separation into seven clusters, where three clusters corresponded to the *Auricomus* subgenus and four clusters to the *Ranunculus* subgenus (Fig 3).

In the *Auricomus* subgenus, *R*. *sceleratus* was the closest taxon to the first cluster consisting of six species from the sections *Hecatonia* and *Batrachium*, where four species of *Batrachium* were confidently separated from species of *Hecatonia*. Notably, *R*. *rionii* Lagger, the species with a previously unknown position in genus taxonomy, was clustered together with *R*. *natans* from the section *Hecatonia*. The second cluster includes 16 taxa of the section *Auricomus*, including four local species (*R*. *songaricus* Schrenk, *R*. *albertii* Regel & Schmalh., *R*. *polyrhizos* Stephan ex Willd., and *R*. *rubrocalyx* Regel ex Kom.). The third cluster consists of two species of the section *Flammula* that was the closest group to representatives of the subgenus *Ranunculus* (Fig 3).

The subgenus *Ranunculus* clade comprises taxa of the sections *Ranunculus*, *Echinella*, *Polyanthemos*, and *Ranunculastrum* (Fig 3). The section *Ranunculus* was the first clustered group in the subgenus after the section *Flammula* and consists of eight taxa, including four species from Kazakhstan. The list of four species includes *R*. *karkaralensis* from Central Kazakhstan, which was previously described as a new species in the genus. The ITS sequence alignment of six taxa that were grouped in the section *Ranunculus* showed that *R*. *acris*, *R*. *grandifolius*, *R*. *subborealis*, and *R*. *karkaralenis* are invariant in all three segments of the ITS (ITS1, 5.8S, and ITS2). This group of species has two nucleotide differences from *R*. *turneri* and five nucleotide differences from *R*. *japonicus* Thunb. (Table 1). As three out of those five polymorphic nucleotides are in ITS2, it is interesting to note that ITS2 is a more informative segment for this subclade, although the total number of polymorphic nucleotides for the entire genus was higher in ITS1 in comparison to ITS2.

**Table 1:** Polymorphic nucleotide sites of *R*. *japonicas*, *R*. *turneri*, *R*. *grandifolius*, *R*. *acris*, *R*. *subborealis*, and *R*. *karkaralenis* in ITS.

ITS	ITS 1	5,8 S	ITS 2				
**Polymorphic site number**	**77**	**134**	**143**	**156**	**172**	**174**	**175**
**Nucleotide position**	**127**	**383**	**417**	**437**	**501**	**516**	**517**
EU591982 *R*. *japonicus*	A	C	G	A	A	C	G
FM242817 *R*. *turneri*	G	-	A	-	-	G	C
MT271831 *R*. subborealis*	G	-	A	-	-	C	G
MT271841 *R*. karkaralensis*	G	-	A	-	-	C	G
MT271832 *R*. grandifolius*	G	-	A	-	-	C	G
AY680167 *R*. *acris*	G	-	A	-	-	C	G

* denotes the species analyzed in this study.

In the next segment of the phylogenetic tree, a solo species *R*. *arvensis* L. in the section *Echinella*, and four species of the section *Polyanthemos* were placed after the section *Ranunculus* (Fig 3). Finally, the last subclade contained 27 specimens, including 20 taxa that were previously assigned to the section *Ranunculastrum*. Therefore, it was hypothesized that seven unassigned species in this subcluster also belong to the section *Ranunculastrum*. Three out of seven unassigned taxa (*R*. *talassicus*, *R*. *alaiensis*, and *R*. *pskemensis*) are species endemic to Central Asia, with two of them (*R*. *talassicus* and *R*. *pskemensis*) growing in southern Kazakhstan (S1 Table). A separate group consisting of these three Central Asian species was analyzed against taxa from eight sections of the subgenera *Auricomus* and *Ranunculus* using Nei’s unbiased genetic distance (Table 2). The results suggest that this group of species is genetically closer to the section *Ranunculastrum* (0.005), followed by sections *Polyanthemos* (0.035) and *Echinella* (0.051), which is an additional indication that all previously unassigned species in this subclade belong to the section *Ranunculastrum*.

**Table 2:** The mean Nei’s genetic distances among samples from eight sections of the genus *Ranunculus*.

	Auricomus (n = 16)	Batrachium (n = 5)	Flammula (n = 2)	Hecatonia (n = 2)	Echinella (n = 2)	Polyanthemos (n = 4)	Ranunculastrum (n = 22)	Ranunculus (n = 8)
*Batrachium*	0.037							
*Flammula*	0.075	0.053						
*Hecatonia*	0.042	0.013	0.057					
*Echinella*	0.106	0.090	0.085	0.087				
*Polyanthemos*	0.090	0.078	0.066	0.079	0.050			
*Ranunculastrum*	0.088	0.080	0.071	0.078	0.049	0.035		
*Ranunculus*	0.109	0.090	0.087	0.087	0.066	0.059	0.052	
UAS*	0.089	0.083	0.073	0.080	0.051	0.035	0.005	0,054

* UAS represents unassigned species and consisted of *R*. *talassicus*, *R*. *alaiensis*, *R*. *pskemensis*, *R*. *breyninus*, and *R*. *ampelophyllus*.

In a separate study, the alignment of ITS sequences from *R*. *talassicus*, *R*. *alaiensis*, and *R*. *pskemensis* with *R*. *illyricus*, which is a key species in the section *Ranunculastrum*, suggested that *R*. *talassicus*is is the most distant species from *R*. *illyricus* (25 base pairs), followed by *R*. *alaiensis* (24 bp) and *R*. *pskemensis* (20 bp) (Table 3). A direct comparison of ITS sequences in *R*. *talassicus* and *R*. *alaiensis* revealed eight nucleotide differences.

**Table 3:** Polymorphic sites of *R*. *illyricus*, *R*. *talassicus*, *R*. *alaiensis*, and *R*. *pskemensis* in ITS.

ITS	ITS 1																	5.8 S	ITS 2													
Polymorphic site number	1	2	3	4	5	6	7	8	9	10	11	12	13	14	15	16	17	18	19	20	21	22	23	24	25	26	27	28	29	30	31	32
Nucleotide position	14	47	58	61	67	94	98	102	106	121	134	145	162	207	208	219	221	358	416	418	423	424	427	434	462	464	470	509	558	562	567	576
AY680119 *R*. *illyricus*	A	G	G	T	C	G	G	-	C	T	C	C	T	C	T	T	T	G	T	C	T	C	-	A	A	T	C	C	-	G	A	T
MT271839 *R*. pskemensis*	T	G	G	C	C	G	A	A	T	T	T	A	C	T	T	C	A	G	A	T	C	T	-	G	T	C	C	C	C	G	A	C
MT271830 *R*. alaiensis*	A	G	G	C	T	A	A	-	T	C	T	T	C	T	C	C	A	G	C	C	C	T	T	G	T	T	T	T	C	G	C	A
MN151383 *R*. talassicus*	A	A	A	C	C	A	A	-	T	C	T	T	C	T	C	C	A	A	C	C	C	T	T	G	T	T	T	C	A	A	A	A

* denotes species analyzed in this study.

In general, Nei’s genetic distance (Table 2) suggested that the nearest sections were *Hecatonia* and *Batrachium* (0.013), and the most distant sections were *Auricomus* and *Ranunculus* (0.109). The analysis of molecular variance (AMOVA) for the ITS sequences of specimens from eight sections suggested that the total genetic variation was partitioned into 73% of the variation within the sections and 27% between sections.

**Fig 4:**
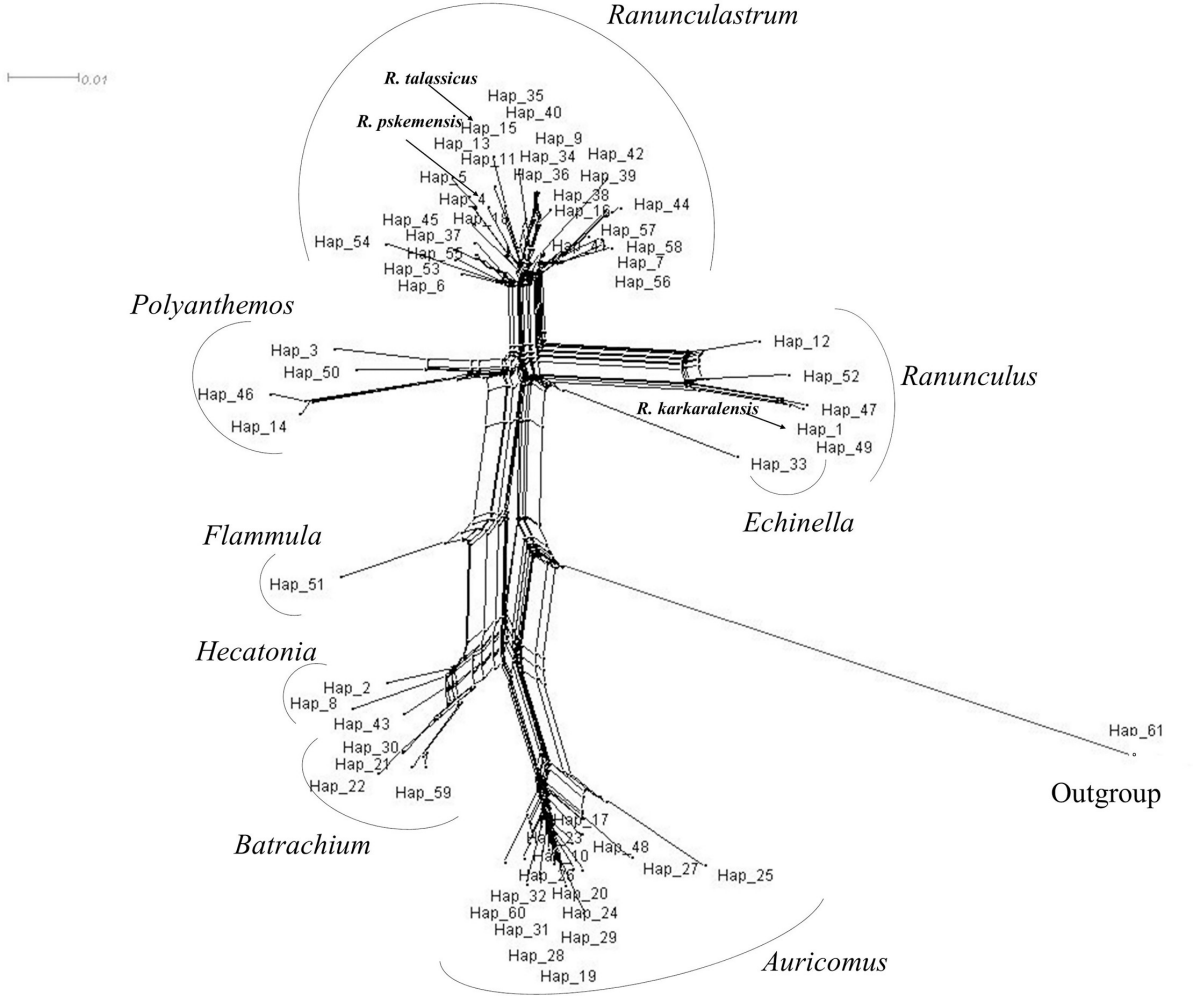
Neighbor-Net graph based on *Ranunculus* ITS sequences.

The second analysis was related to haplotype network assessment, where nucleotide sequences were converted into haplotypes. In total, the network reconstruction allowed the identification of 61 haplotypes, including outgroups (Fig 4, S2 Table).

The haplotype diversity for this set of data, Hd, was 0.996; the nucleotide diversity, π, was 0.064; and the average number of nucleotide differences, k, was 33.033. The haplotype groups generated corresponded to the MP phylogenetic tree subclades (Fig 3), and the profile of the network confirmed the monophyletic origin of the genus. The network, with outgroups, confirmed the separation of the sections in the subgenera *Auricomus* and *Ranunculus*. The *Auricomus* profile hinted that species from the sections *Hecatonia* and *Batrachium* are closest to the most common predecessors of the genus, as their profiles were next to the nodes, which linked directly with the outgroups (Fig 4). Notably, *R*. *rionii* (Hap 43, Gene bank accession FM242855), which had previously not been studied in the genus taxonomy, was positioned in the middle between *Hecatonia* and *Batrachium* species, unlike its position in the phylogenetic tree (Fig 3). The subgenera *Ranunculus* profile of the network is nearly congruent with the phylogenetic tree, except for the section *Echinella*, which, similar to *Hecatonia* and *Batrachium*, is located closer to the hypothetical most common evolutionary ancestor (MCEA) of the genus from the opposite side of the subgenera *Auricomus* (Fig 4). Further in the network, in the section *Ranunculus*, the key haplotype is Hap 1, which not only consists of the four invariable species *R*. *grandifolius*, *R*. *acris*, *R*. *subborealis*, and *R*. *karkaralensis*, but is also close to Hap 47 (*R*. *turneri*) and Hap 49 (*R*. *japonicus*) (Fig 4). In the section *Ranunculastrum*, the network profile indicates that the first haplotypes linked with the median vector on the main axis were Hap 6 (*R*. *pedatus*) and Hap 54 (*R*. *illyricus*). The network confirms that haplotype Hap 15 of the newly discovered species *R*. *talassicus* is connected to Hap 11 (*R*. *alaiensis*). The branch with these two species has a root with *R*. *paucidentatus* (Hap 18). A larger group of branches containing ten species, including *R*. *talassicus*, has a root with a more ancient node connected with *R*. *pskemensis* (Hap 4).

## Discussion

The taxonomic order in the study was determined using 226 species of the genus and by the application of DNA markers and morphological data. In the current study, the molecular phylogeny of three Central Asian endemic species, *R*. *karkaralensis*, *R*. *talassicus*, and *R*. *pskemensis* was assessed using 65 *Ranunculus* species, including 43 taxa from NCBI, two outgroup species, and ITS nuclear genome marker data. The analysis of polymorphism levels in the ITS indicated that for 591 aligned ITS nucleotides, 206 bases were polymorphic, including 121 bases in ITS1, 14 in 5.8S, and 71 in ITS2. Recently, two reports have discussed the level of variability in different DNA barcoding markers for the differentiation of medicinal plants [46, 47]. The authors suggested that ITS2 is one of the most appropriate markers for species identification and is more informative than other types of markers, including ITS1. However, the results in this study indicate that the number of polymorphic sites in ITS1 is higher than that in ITS2. Hence, the outcome suggests that it is useful and possibly necessary to use both ITS1 and ITS2 spacers for phylogenetic studies.

Despite the smaller number of samples and lower bootstrap values in the current study in comparison with the report of Hörandl and Emadzade (2012) [14], the profiles generated in the phylogenetic trees were similar. The profiles of both the MP phylogenetic tree and the Neighbor-Net network within the present study were also nearly congruent (Figs 3 and 4). Therefore, these similarities suggest taxonomic assessment of these three endemic species is reliable. However, there were minor differences in positions between the phylogenetic tree and the Neighbor-Net network. These differences include the closer distance of the section *Echinella* from the edge of subgenus *Ranunculus* to the MCEA, unlike the result from the phylogenetic tree, where *Ranunculus* is looked as early-diverging section in the subgenus (Fig 3).

The intragenera differences of taxa identified by the Neighbor-Net network for this group of sections were most probably predetermined by species evolution, genetic distinction, geographic distribution, and ecological conditions of growth [14]. For instance, the section *Ranunculus* consists of approximately 30, mostly perennial, species, both diploid and polyploid, widely distributed in Eurasia, North America, and Africa, with the basal number of chromosomes x = 7 (rarely 8) [14]. The section *Echinella* is rather monotypic and includes *R*. *arvensis*, which is an annual species with enlarged nuts that have a size of more than 5 mm. *R*. *arvensis* is a very cosmopolitan species with a wide distribution in Eurasia and is characterized by the basal number of chromosomes x = 8 [14]. The section *Polyanthemos* is the largest group (80–90 species); is very morphologically diverse; has both annual and perennial species; has diploids and polyploids with the basal number of chromosomes x = 8 (rarely 7); and is spread over all continents, except Antarctica [9, 48]. The phylogenetic studies suggest that *Polyanthemos* includes 46 species from the Southern Hemisphere, with regional clades formed in Australia, Malaysia, and South America [9, 14, 48]. These findings may explain the phylogenetic position of the section *Polyanthemos*, which contrasts with the positions of *Ranunculus* and *Echinella* (Figs 3 and 4), since the origin of these two sections is associated with the Eurasian continent.

The similarities of phylogeny between sections *Batrachium* and *Hecatonia*, shown in Figs 3 and 4, can presumably be linked to commonalities associated with aquatic and wetland conditions of growth. Previously, species of the section *Batrachium* were often classified as a separate genus or subgenus. The section is well-spread over all continents, except Antarctica, and is composed of annual and perennial species with white-flowered buttercups and sometimes with a high ability for cross-hybridization [49]. A poor clusterization of *R*. *sceleratus* and *R*. *natans* in the section *Hecatonia* (Fig 3) supported the observation that this section is more morphologically diverse than *Batrachium* [14]. The species in *Hecatonia* are mostly distributed in the Northern Hemisphere and also in South America and Antarctica. *Hecatonia* is represented by annual and perennial species and diploids and polyploids, with yellow flowers and well-developed stem leaves [14]. Hence, the clustering of species in these two sections may reflect visible morphological differences and geo-ecological conditions of growth.

The evaluation of the ITS nucleotide sequence in *R*. *karkaralensis* suggested no difference from *R*. *acris*, *R*. *grandifolius*, and *R*. *subborealis*, and all of these taxa were placed in the section *Ranunculus*. Morphologically, *R*. *karkaralensis* is the most close to *R*. *grandifolius*, as they only differ in terms of the absence of a well-developed horizontal rhizome and the shape and proportions of the leaf blade of the basal leaves. The lack of differences in the ITS region for these species could be explained by several reasons, such as (i) the low-resolution power of the selected marker for these species, (ii) recent speciation events in the newly identified species, and (iii) high hybridization rates among subspecies within a species. Therefore, despite obvious morphological differences between *R*. *grandifolius* and *R*. *karkaralensis* [26], additional evidence is likely to be required to accept the latter as a separate species. A very different outcome was revealed in the genetic analyses of *R*. *talassicus* and *R*. *pskemensis* in comparison with other taxa of the genus. The assessment of the genetic distances between species (Table 2) and the phylogenetic tree (Fig 3) clearly placed *R*. *talassicus* and *R*. *pskemensis* in the section *Ranunculastrum* within the subgenus *Ranunculus* (Fig 4). The phylogenetic tree, similar to the network, clustered *R*. *talassicus* together with *R*. *alaiensis* (Figs 3 and 4). The result supports the botanical assessment, which suggests strong similarities between these vicar species [27]. Morphologically, *R*. *talassicus* is very similar to *R*. *alaiensis*; however, these species are easily distinguished by the shape of the basal leaves. In addition to geographical fragmentation, they grow at different altitudes, although the ecological niches are similar. *R*. *talassicus* is found in the Western Tien-Shan at an altitude of 2400–3200 m above sea level. *R*. *alaiensis* is found in the high mountains of the Pamir-Alai, and its altitudinal confinement begins at 2700 m on the Darvaz Range and 3500 m above sea level on the Gissar Range. *R*. *talassicus* is also morphologically similar to *R*. *pskemensis*, and the latter species is also found in the western part of the Tien-Shan. However, unlike *R*. *talassicus*, *R*. *pskemensis* occupies other habitats [27]. The ITS sequence alignment revealed that *R*. *talassicus* was 20 nucleotide bases away from *R*. *pskemensis* and 25 bases from *R*. *illyricus* (Table 3). The difference was reflected in the network, with *R*. *pskemensis* forming the root for the larger branch with *R*. *talassicus*, and *R*. *illyricus* along with *R*. *pedatus* forming a prime rooting node for the section (Fig 4). Therefore, the taxonomic positions for both Central Asian endemic species *R*. *talassicus* and *R*. *pskemensis* are well defined.

## Conclusions

*Ranunculus* is a very large and complex genus spread across moderately cold climatic regions worldwide. Studies of its molecular taxonomy have shown that this genus is well-classified and suggest a separation of 226 species into two subgenera and 17 sections. However, despite a thorough botanical classification of taxa in the genus, the molecular taxonomy of endemic species from Central Asia is poorly understood. Therefore, three previously unstudied Kazakh species, along with 19 other local species and 43 taxa from the NCBI, were analyzed using the ITS marker region of the nuclear genome. The analysis of ITS sequences confirmed the monophyletic origin of the genus and clustered the previously unstudied Central Asian species into their sections. It was determined that *R*. *karkaralensis* has invariant ITS sequences with three other species in the section *Ranunculus*, i.e., *R*. *acris*, *R*. *grandifolius*, and *R*. *subborealis*. The two other endemic Central Asian species *R*. *talassicus*, and *R*. *pskemensis* were assigned to the section *Ranunculastrum*. It was revealed that *R*. *talassicus* is most closely related to *R*. *alaiensis*. Therefore, the application of ITS-aligned sequences has provided new insights into the taxonomic positions of three endemic species from Central Asia.

## Supporting information

S1 Table Geographic locations of the sampling sites. (DOCX)

S2 Table The list of haplotypes from the analysis of ITS sequences of Ranunculus. Species and outgroups.
(DOCX)

S1 Appendix Permission letter from the Sairam-Ugam Natrional Nature Park. (PDF)

S2 Appendix Phylogenetic trees generated by using Neighbor Joining (NJ), Maximum Likelihood (ML), and Maximum Parsimony (MP) methods, with bootstrap of 100, 500, 1000, 5000, 10000 replicates. (PDF)
