# Supplementary material for: The molecular taxonomy of three endemic Central Asian species of Ranunculus(Ranunculaceae)
Source: PLoS One. 2020 Oct 5;15(10):e0240121. doi: 10.1371/journal.pone.0240121 (PMC7535031; doi:10.1371/journal.pone.0240121)
Supplement: S1 Table — (DOCX) [file pone.0240121.s001.docx]

**S1 Table.** Geographic locations of the sampling sites

| # | NCBI accession number | Species | Collected date | Collected place | Collector |
| --- | --- | --- | --- | --- | --- |
| 1 | MN151375 | *R. rubrocalyx* | 29.05.2018 | Kazakhstan, Turkestan region. Sayram-Ugam National Park | Natalia V. Shchegoleva |
| 2 | MN151376 | *R. rubrocalyx* | 29.05.2018 | Kazakhstan, Turkestan region. Sayram-Ugam National Park | Natalia V. Shchegoleva |
| 3 | MN151377 | *R. songaricus* | 28.05.2018 | Kazakhstan, Turkestan region. Sayram-Ugam National Park | Natalia V. Shchegoleva |
| 4 | MN151378 | *R. songaricus* | 29.05.2018 | Kazakhstan, Turkestan region. Sayram-Ugam National Park | Natalia V. Shchegoleva |
| 5 | MN151379 | *R. paucidentatus* | 29.05.2018 | Kazakhstan, Turkestan region. Sayram-Ugam National Park | Natalia V. Shchegoleva |
| 6 | MN151380 | *R. polyrhizos* | 28.05.2018 | Kazakhstan, Turkestan region. Sayram-Ugam National Park | Natalia V. Shchegoleva |
| 7 | MN151383 | *R. talassicus* | 28.05.2018 | Kazakhstan, Turkestan region. Sayram-Ugam National Park | Natalia V. Shchegoleva |
| 8 | MN151387 | *R. regelianus* | 29.05.2018 | Kazakhstan, Turkestan region. Sayram-Ugam National Park | Natalia V. Shchegoleva |
| 9 | MN151381 | *R. olgae* | 28.05.2018 | Kazakhstan, Turkestan region. Sayram-Ugam National Park | Natalia V. Shchegoleva |
| 10 | MN151382 | *R. olgae* | 28.05.2018 | Kazakhstan, Turkestan region. Sayram-Ugam National Park | Natalia V. Shchegoleva |
| 11 | MN151388 | *R. laetus* | 30.05.2018 | Kazakhstan, Turkestan region. Sayram-Ugam National Park | Natalia V. Shchegoleva |
| 12 | MN151384 | *R. polyanthemos* | 30.05.2018 | Kazakhstan, Turkestan region. Sayram-Ugam National Park | Natalia V. Shchegoleva |
| 13 | MN151385 | *R. polyanthemos* | 2015 | Kazakhstan, Pavlodar region, Bayanaul | Anna P. Muranets |
| 14 | MN151386 | *R. polyanthemos* |  |  |  |
| 15 | MT271830 | *R. alaiensis* | 15.06.2019 | Tajikistan, Darvaz Range | Natalia V. Shchegoleva |
| 16 | MT271831 | *R. subborealis* | 25.05.2008 | Kazakhstan, Karaganda region | Natalia V. Shchegoleva |
| 17 | MT271832 | *R. grandifolius* | 24.05.2008 | Kazakhstan, Karaganda region | Natalia V. Shchegoleva |
| 18 | MT271833 | *R. natans* | 14.06.1998 | Kazakhstan, East Kazakhstan region, Saykan ridge | Olga M. Maslova,  Irina A. Khrustaleva,  Nadezhda A. Usik,  Tatyana O. Strelnikova |
| 19 | MT271834 | *R. oxyspermus* | 16.04.2014 | Kazakhstan, South Kazakhstan Region. Baidibek District, Karatau Range / Boroldai Gorge | Andrey N. Kupriyanov |
| 20 | MT271835 | *R. repens* | 24.05.2010 | Kazakhstan, Karaganda region, Nurinsky district, river bank Kerey | Natalia V. Shchegoleva |
| 21 | MT271836 | *R. sceleratus* | 22.05.2010 | Kazakhstan, Karaganda region, Ulytau Mountains. | Natalia V. Shchegoleva |
| 22 | MN151380 | *R. polyrhizos* | 13.05.2010 | Kazakhstan, Karaganda region, Bukhara-Zhirau region. Spassky hills | Natalia V. Shchegoleva |
| 23 | MT271837 | *R. pedatus* | 14.05.2010 | Kazakhstan, Karaganda region, village of Akkoy | Natalia V. Shchegoleva |
| 24 | MT271838 | *R. platyspermus* | 09.04.2019 | Kazakhstan, Mangystau district | Nikolay N.  Lashchinskiy |
| 25 | NA | *R. platyspermus* | 18.05.2010 | Kazakhstan, Karaganda region, north Betpakdala | Natalya V. Shchegoleva |
| 26 | MT271839 | *R. pskemensis* | 05.07.2019 | Kazakhstan, Turkestan region. Sayram-Ugam National Park | Irina A. Khrustaleva,  Alexander L. Ebel |
| 27 | MT271840 | *R. albertii* | 16.08.2017 | Kazakhstan, Trans-Ili Alatau mountains | Natalia V. Shchegoleva |
| 28 | MT271841 | *R. karkaralensis* | 24.05.2008 | Kazakhstan, Karaganda region. Karkaraly Mountains, at the foot of the Zhiren-Sakal Mountain | Natalia V. Shchegoleva |
| 29 | MT271842 | *R. linearilobus* | 13.04.2018 | Uzbekistan East Kuldzhiktau | Andrey Yu. Korolyuk |
