# Supplementary material for: The molecular taxonomy of three endemic Central Asian species of Ranunculus(Ranunculaceae)
Source: PLoS One. 2020 Oct 5;15(10):e0240121. doi: 10.1371/journal.pone.0240121 (PMC7535031; doi:10.1371/journal.pone.0240121)
Supplement: S2 Table — Species and outgroups. (DOCX) [file pone.0240121.s002.docx]

**S2 Table.** Haplotypes formed from the analysis of ITS sequences of *Ranunculus* species and outgroups

| **Haplo**  **type** | **Number of species** | **Species** | **GenBank Accession number** | **Section** | **Subgenus** |
| --- | --- | --- | --- | --- | --- |
| Hap 1 | 4 | *Ranunculus subborealis** | MT271831 | *Ranunculus* | *Ranunculus* |
|  |  | *Ranunculus karkaralensis** | MT271841 | *Ranunculus* | *Ranunculus* |
|  |  | *Ranunculus grandifolius** | MT271832 | *Ranunculus* | *Ranunculus* |
|  |  | *Ranunculus acris* | AY680167 | *Ranunculus* | *Ranunculus* |
| Hap 2 | 1 | *Ranunculus sceleratus** | MT271836 | *Hecatonia* | *Auricomus* |
| Hap 3 | 1 | *Ranunculus repens** | MT271835 | *Polyanthemos* | *Ranunculus* |
| Hap 4 | 1 | *Ranunculus pskemensis** | MT271839 | *-* | *-* |
| Hap 5 | 1 | *Ranunculus platyspermus** | MT271838 | *Ranunculastrum* | *Ranunculus* |
| Hap 6 | 1 | *Ranunculus pedatus** | MT271837 | *Ranunculastrum* | *Ranunculus* |
| Hap 7 | 1 | *Ranunculus oxyspermus** | MT271834 | *Ranunculastrum* | *Ranunculus* |
| Hap 8 | 1 | *Ranunculus natans** | MT271833 | *Hecatonia* | *Auricomus* |
| Hap 9 | 1 | *Ranunculus linearilobus** | MT271842 | *Ranunculastrum* | *Ranunculus* |
| Hap 10 | 2 | *Ranunculus albertii** | MT271840 | *Auricomus* | *Auricomus* |
| Hap 11 | 1 | *Ranunculus alaiensis** | MT271830 | *-* | *-* |
| Hap 12 | 1 | *Ranunculus laetus** | MN151388 | *Ranunculus* | *Ranunculus* |
| Hap 13 | 1 | *Ranunculus regelianus** | MN151387 | *Ranunculastrum* | *Ranunculus* |
| Hap 14 | 1 | *Ranunculus polyanthemos** | MN151384 | *Polyanthemos* | *Ranunculus* |
| Hap 15 | 1 | *Ranunculus talassicus** | MN151383 | *-* | *-* |
| Hap 16 | 3 | *Ranunculus olgae** | MN151381 | *-* | *-* |
| Hap 17 | 1 | *Ranunculus polyrhizos** | MN151380 | *Auricomus* | *Auricomus* |
| Hap 18 | 1 | *Ranunculus paucidentatus** | MN151379 | *Ranunculastrum* | *Ranunculus* |
| Hap 19 | 1 | *Ranunculus songaricus** | MN151377 | *Auricomus* | *Auricomus* |
| Hap 20 | 1 | *Ranunculus rubrocalyx** | MN151375 | *Auricomus* | *Auricomus* |
| Hap 21 | 1 | *Ranunculus kaufanii* | MG098949 | *Batrachium* | *Auricomus* |
| Hap 22 | 1 | *Ranunculus mongolicus* | KR996527 | *Batrachium* | *Auricomus* |
| Hap 23 | 1 | *Ranunculus pygmaeus* | KP687287 | *Auricomus* | *Auricomus* |
| Hap 24 | 1 | *Ranunculus pulchellus* | KP687285 | *Auricomus* | *Auricomus* |
| Hap 25 | 1 | *Ranunculus jovis* | KP687281 | *Auricomus* | *Auricomus* |
| Hap 26 | 1 | *Ranunculus inamoenus* | KP687279 | *Auricomus* | *Auricomus* |
| Hap 27 | 1 | *Ranunculus glaberrimus* | KP687273 | *Auricomus* | *Auricomus* |
| Hap 28 | 2 | *Ranunculus brotherusii* | KP687272 | *Auricomus* | *Auricomus* |
|  |  | *Ranunculus pegaeus* | JF509967 | *Auricomus* | *Auricomus* |
| Hap 29 | 1 | *Ranunculus adoneus* | KP687265 | *Auricomus* | *Auricomus* |
| Hap 30 | 1 | *Ranunculus trichophyllus* | KC620483 | *Batrachium* | *Auricomus* |
| Hap 31 | 1 | *Ranunculus sulphureus* | JF509969 | *Auricomus* | *Auricomus* |
| Hap 32 | 1 | *Ranunculus palmatifidus* | JF509966 | *Auricomus* | *Auricomus* |
| Hap 33 | 1 | *Ranunculus arvensis* | HQ650550 | *Echinella* | *Ranunculus* |
| Hap 34 | 2 | *Ranunculus termei* | HQ338346 | *Ranunculastrum* | *Ranunculus* |
| Hap 35 | 1 | *Ranunculus macropodoides* | HQ338326 | *Ranunculastrum* | *Ranunculus* |
| Hap 36 | 1 | *Ranunculus leptorrhynchus* | HQ338318 | *Ranunculastrum* | *Ranunculus* |
| Hap 37 | 1 | *Ranunculus hierosolymitanus* | HQ338318 |  |  |
| Hap 38 | 1 | *Ranunculus elbrusensis* | HQ338311 | *Ranunculastrum* | *Ranunculus* |
| Hap 39 | 1 | *Ranunculus damascenus* | HQ338309 | *Ranunculastrum* | *Ranunculus* |
| Hap 40 | 1 | *Ranunculus aucheri* | HQ338301 | *Ranunculastrum* | *Ranunculus* |
| Hap 41 | 1 | *Ranunculus afghanicus* | HQ338297 | *Ranunculastrum* | *Ranunculus* |
| Hap 42 | 1 | *Ranunculus asiaticus* | GU257963 | *Ranunculastrum* | *Ranunculus* |
| Hap 43 | 1 | *Ranunculus rionii* | FM242855 | *-* | *-* |
| Hap 44 | 1 | *Ranunculus argyrs* | FM242844 | *Ranunculastrum* | *Ranunculus* |
| Hap 45 | 1 | *Ranunculus ampelophyllus* | FM242842 | *-* | *-* |
| Hap 46 | 1 | *Ranunculus submarginatus* | FM242841 | *Polyanthemos* | *Ranunculus* |
| Hap 47 | 1 | *Ranunculus turneri* | FM242817 | *Ranunculus* | *Ranunculus* |
| Hap 48 | 1 | *Ranunculus auricomus* | FM242803 | *Auricomus* | *Auricomus* |
| Hap 49 | 1 | *Ranunculus japonicus* | EU591982 | *Ranunculus* | *Ranunculus* |
| Hap 50 | 1 | *Ranunculus muricatus* | DQ410718 | *Polyanthemos* | *Ranunculus* |
| Hap 51 | 2 | *Ranunculus flammula* | AY680185 | *Flammula* | *Auricomus* |
|  |  | *Ranunculus lingua* | AY680184 | *Flammula* | *Auricomus* |
| Hap 52 | 1 | *Ranunculus baldshuanicus* | AY680174 | *Ranunculus* | *Ranunculus* |
| Hap 53 | 1 | *Ranunculus gracilis* | AY680120 | *Ranunculastrum* | *Ranunculus* |
| Hap 54 | 1 | *Ranunculus illyricus* | AY680119 | *Ranunculastrum* | *Ranunculus* |
| Hap 55 | 1 | *Ranunculus breyninus* | AY680116 | *-* | *-* |
| Hap 56 | 1 | *Ranunculus psilostachys* | AY680106 | *Ranunculastrum* | *Ranunculus* |
| Hap 57 | 1 | *Ranunculus sprunerianus* | AY680105 | *Ranunculastrum* | *Ranunculus* |
| Hap 58 | 1 | *Ranunculus rumelicus* | AY680104 | *Ranunculastrum* | *Ranunculus* |
| Hap 59 | 1 | *Ranunculus sphaerospermus* | AY680066 | *Batrachium* | *Auricomus* |
| Hap 60 | 1 | *Ranunculus rufosepalus* | AY680047 | *Auricomus* | *Auricomus* |
| Hap 61 | 2 | *Trollius altaicus** | MN151394 | Outgroup | - |
|  |  | *Trollius ledebourii* | AY365383 | Outgroup | - |

*– denotes species analyzed in this study
