# Supplementary material for: The molecular taxonomy of three endemic Central Asian species of Ranunculus(Ranunculaceae)
Source: PLoS One. 2020 Oct 5;15(10):e0240121. doi: 10.1371/journal.pone.0240121 (PMC7535031; doi:10.1371/journal.pone.0240121)
Supplement: S1 Appendix — (PDF) [file pone.0240121.s003.pdf]

**ҚАЗАҚСТАН РЕСПУБЛИКАСЫ
ЭКОЛОГИЯ, ГЕОЛОГИЯ ЖӘНЕ ТАБИҒИ РЕСУРСТАР МИНИСТРЛІГІ ОРМАН ШАРУАШЫЛЫҒЫ ЖӘНЕ ЖАНУАРЛАР ДҮНИЕСІ КОМИТЕТІНІҢ «САЙРАМ-ӨГЕМ МЕМЛЕКЕТТІК ҰЛТТЫҚ ТАБИҒИ ПАРКІ» РЕСПУБЛИКАЛЫҚ МЕМЛЕКЕТТІК МЕКЕМЕСІ**

160011, Шымкент қаласы, Г.Иляев көшесі, 24/1
Тел.: 21-28-87 тел/факс 21-27-52
E-mail: sayram_ugam@mail.ru

РЕСПУБЛИКАНСКОЕ ГОСУДАРСТВЕННОЕ УЧРЕЖДЕНИЕ «САЙРАМ-УГАМСКИЙ ГОСУДАРСТВЕННЫЙ НАЦИОНАЛЬНЫЙ ПРИРОДНЫЙ ПАРК» КОМИТЕТА ЛЕСНОГО ХОЗЯЙСТВА И ЖИВОТНОГО МИРА МИНИСТЕРСТВА ЭКОЛОГИИ, ГЕОЛОГИИ И ПРИРОДНЫХ РЕСУРСОВ РЕСПУБЛИКИ КАЗАХСТАН

160011, город Шымкент, ул. Г.Иляев, 24/1
Тел.: 21-28-87 тел/факс 21-27-52
E-mail: sayram_ugam@mail.ru

24.05.2018 № N 844

To: General Director
of the Institute of Plant
Biology and Biotechnology
K. Zhambakin

In response to your letter N145/06 from 02.04.2018, we notify you that scientists from the Institute of Plant Biology and Biotechnology (Almaty, Kazakhstan) and Dr. Natalia Shchegoleva (Tomsk State University, Tomsk, Russia) were permitted to conduct research investigations and collections on the territory of Saigam-Ugam National Nature Park from 26.05.2018 to 31.05.2018.

General Director
of the Saigam-Ugam
National Nature Park

Kyntaev S.Zh.

Сериялық нөмірсіз бланк ЖАРАМСЫЗ ДЕП ТАНЫЛАДЫ. Без серийного номера бланк НЕДЕЙСТВИТЕЛЕН
