# Supplementary material for: The molecular taxonomy of three endemic Central Asian species of Ranunculus(Ranunculaceae)
Source: PLoS One. 2020 Oct 5;15(10):e0240121. doi: 10.1371/journal.pone.0240121 (PMC7535031; doi:10.1371/journal.pone.0240121)

S2 Appendix. Phylogenetic trees generated by using Neighbour Joining (NJ), Maximum Likelihood (ML), and Maximum Parsimony (MP) methods, with bootstrap 100, 500, 1000, 5000, 10000 replicates

NJ tree, 100 replicates

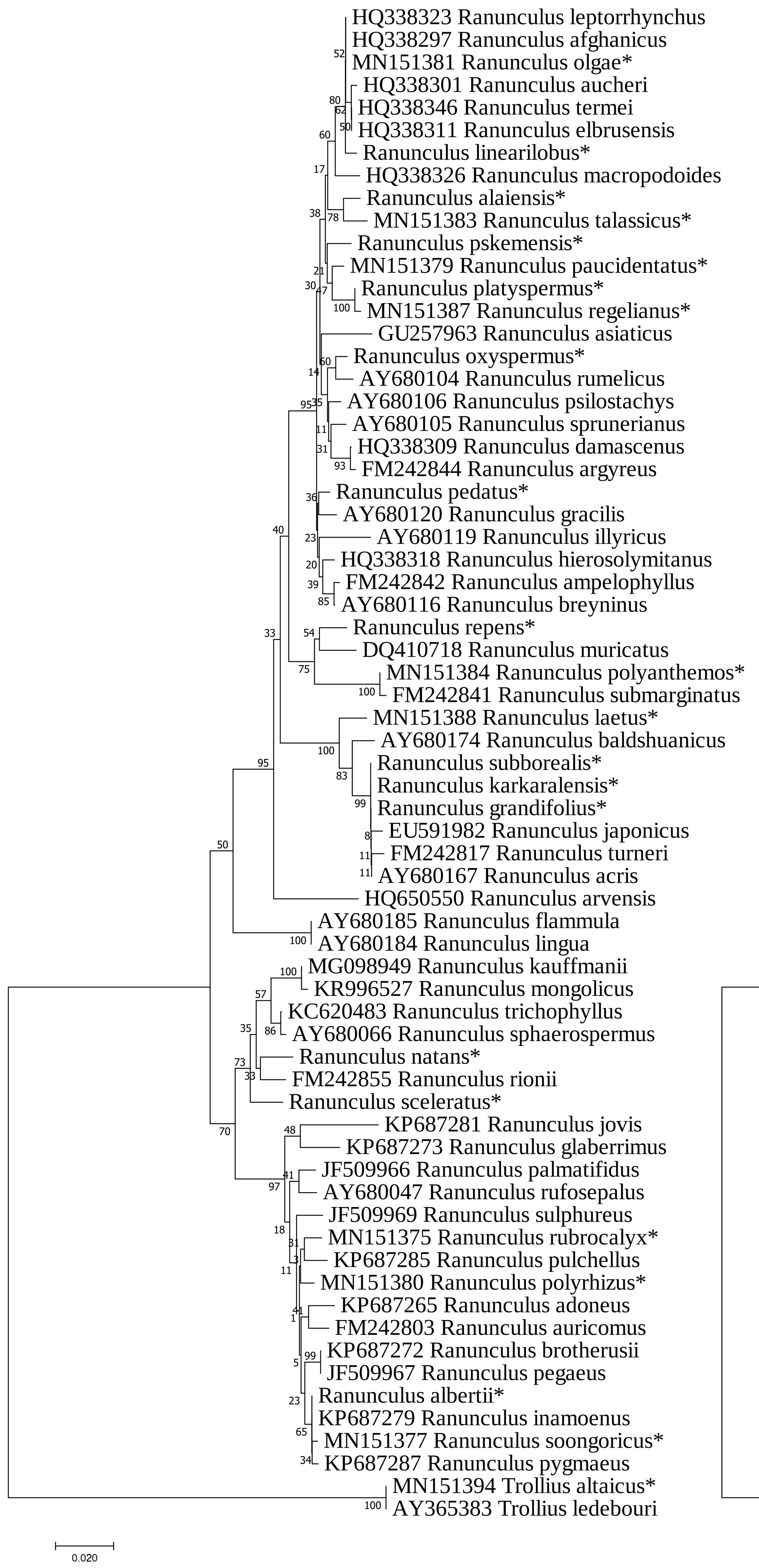

NJ tree, 500 replicates

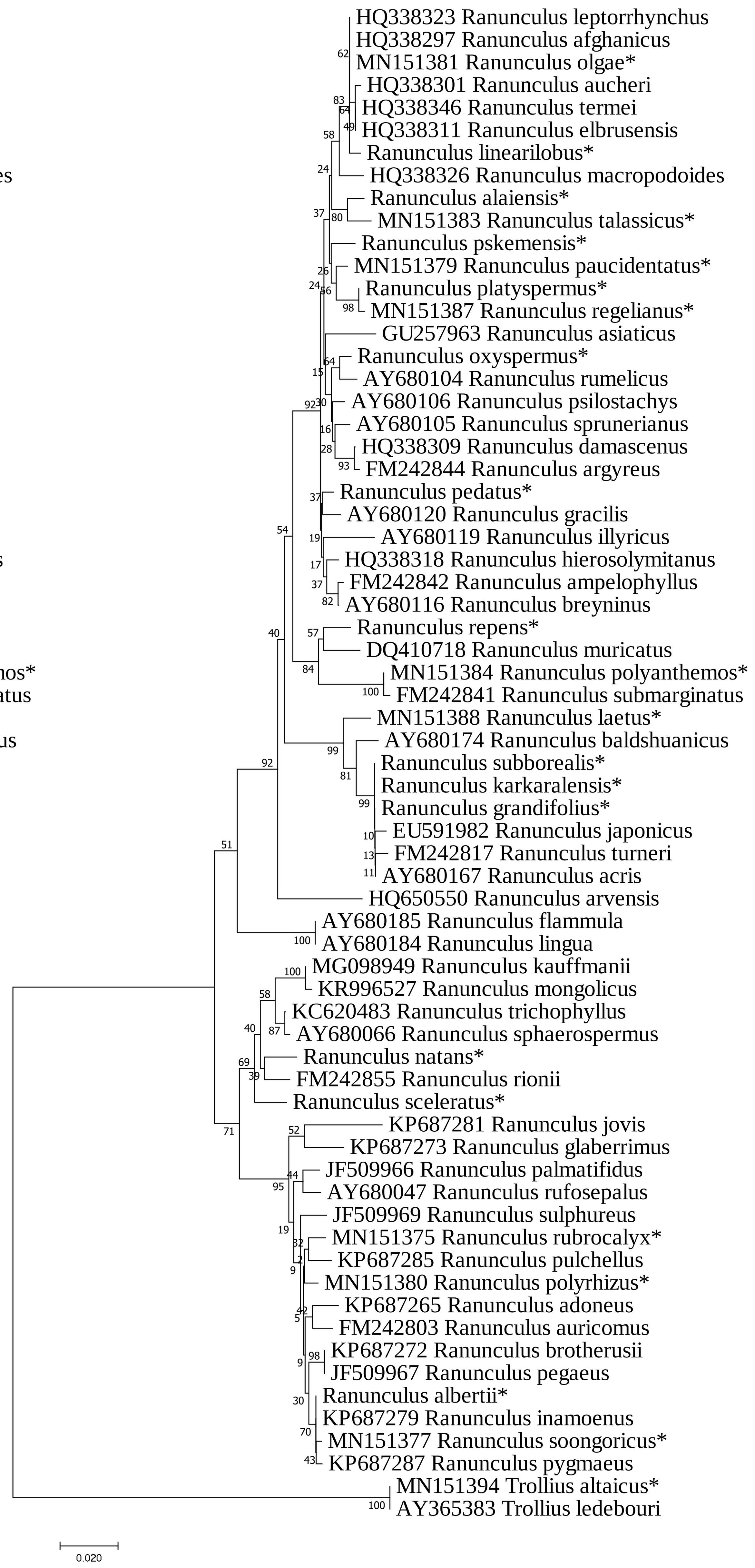

NJ tree, 1000 replicates

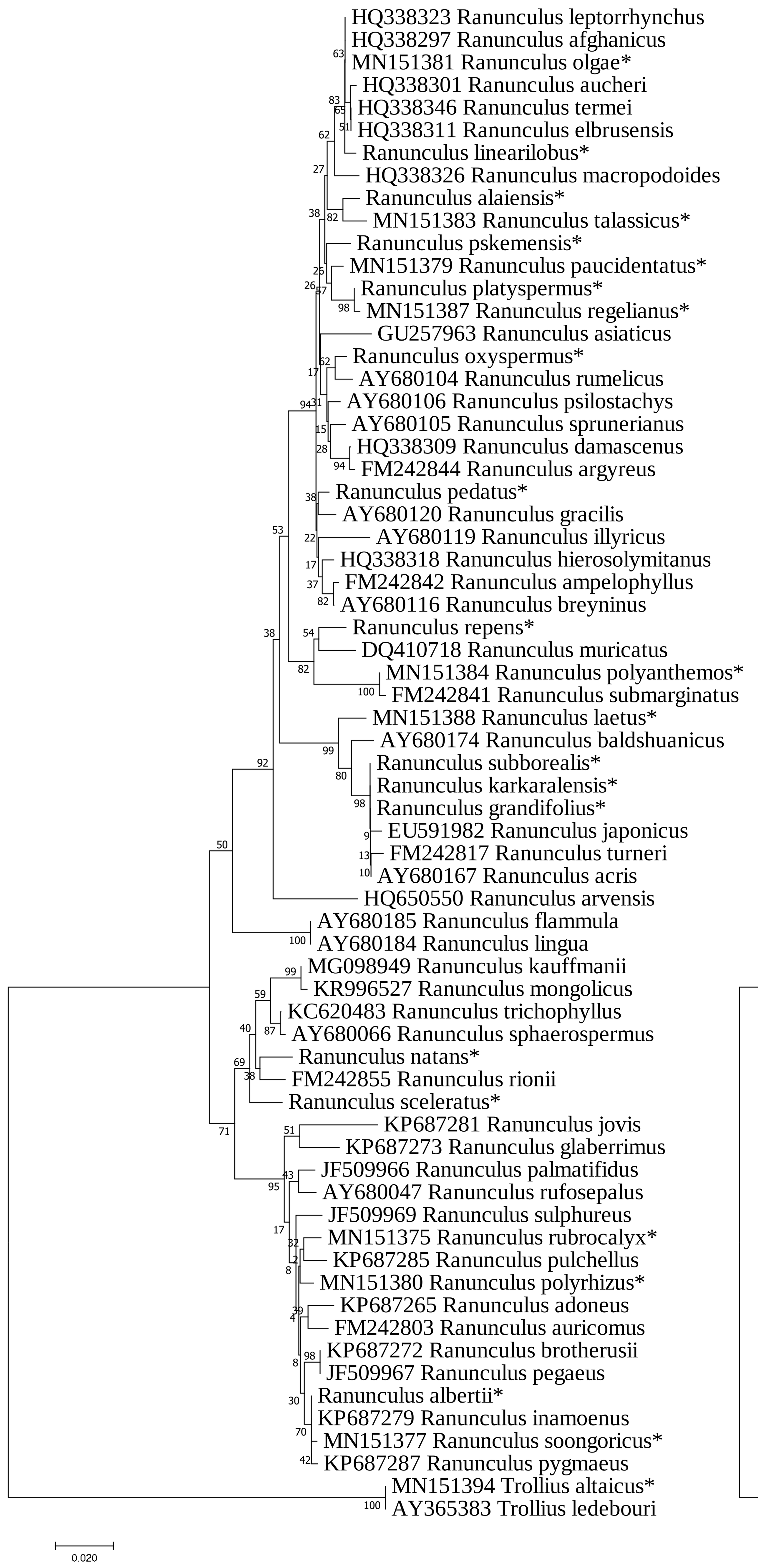

NJ tree, 5000 replicates

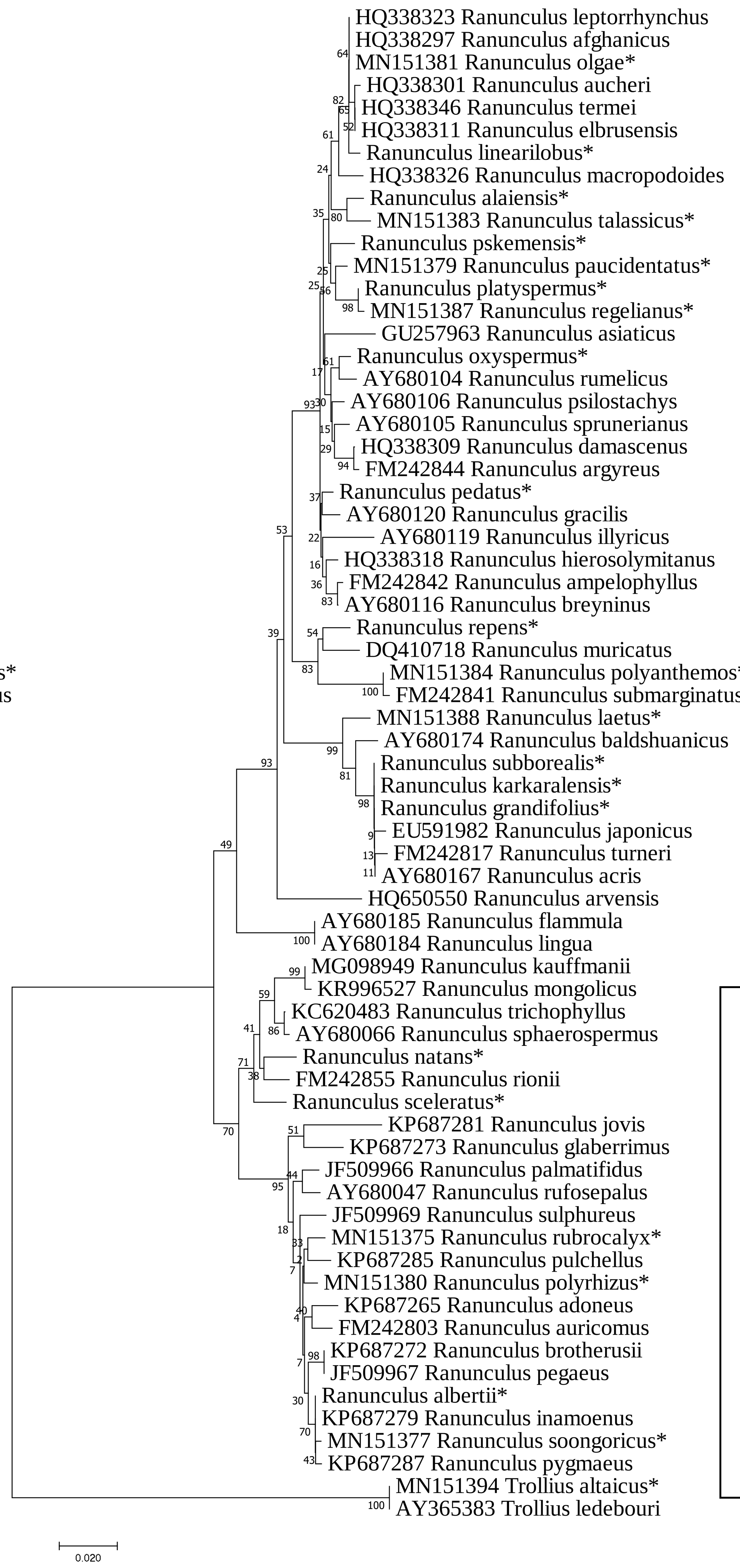

NJ tree, 10 000 replicates

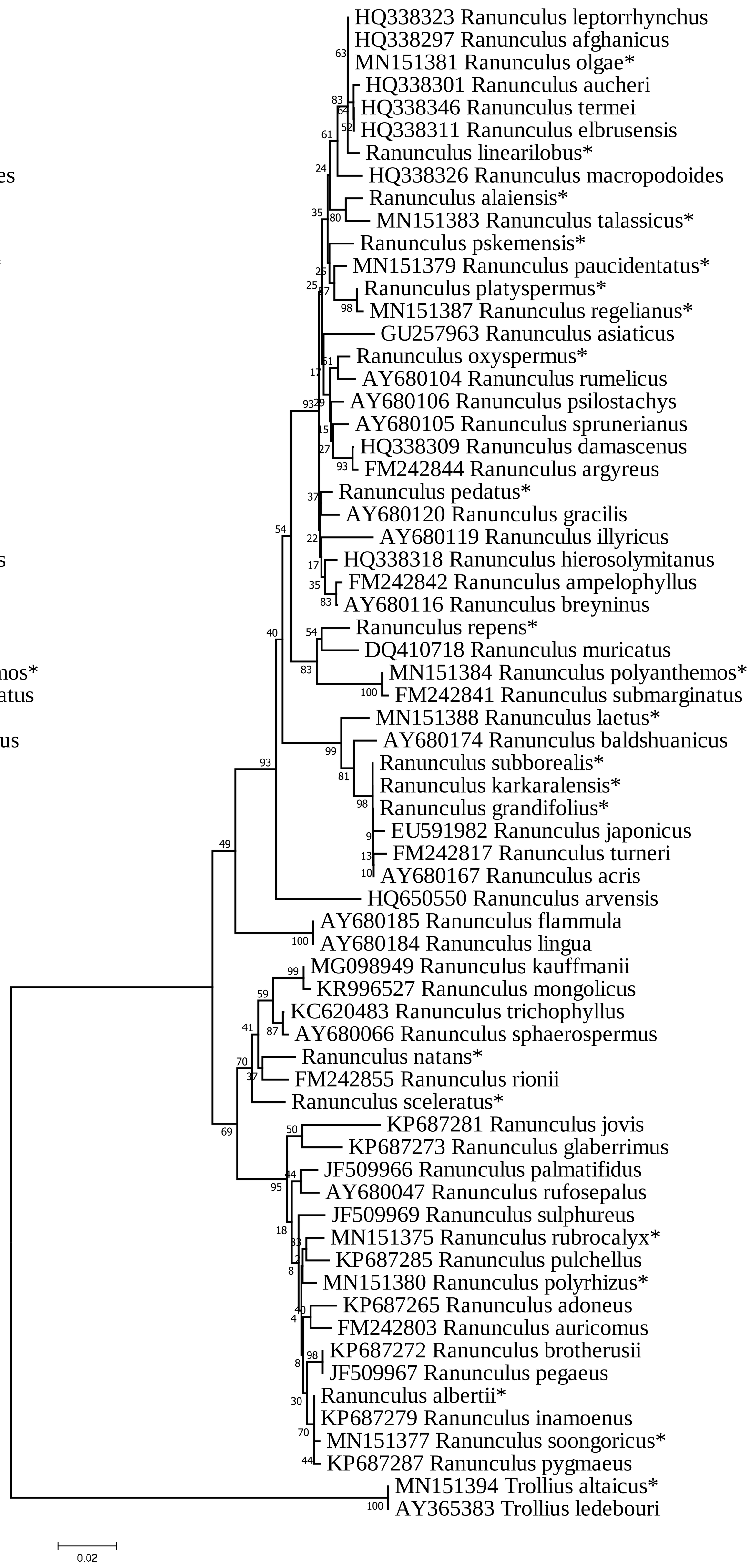

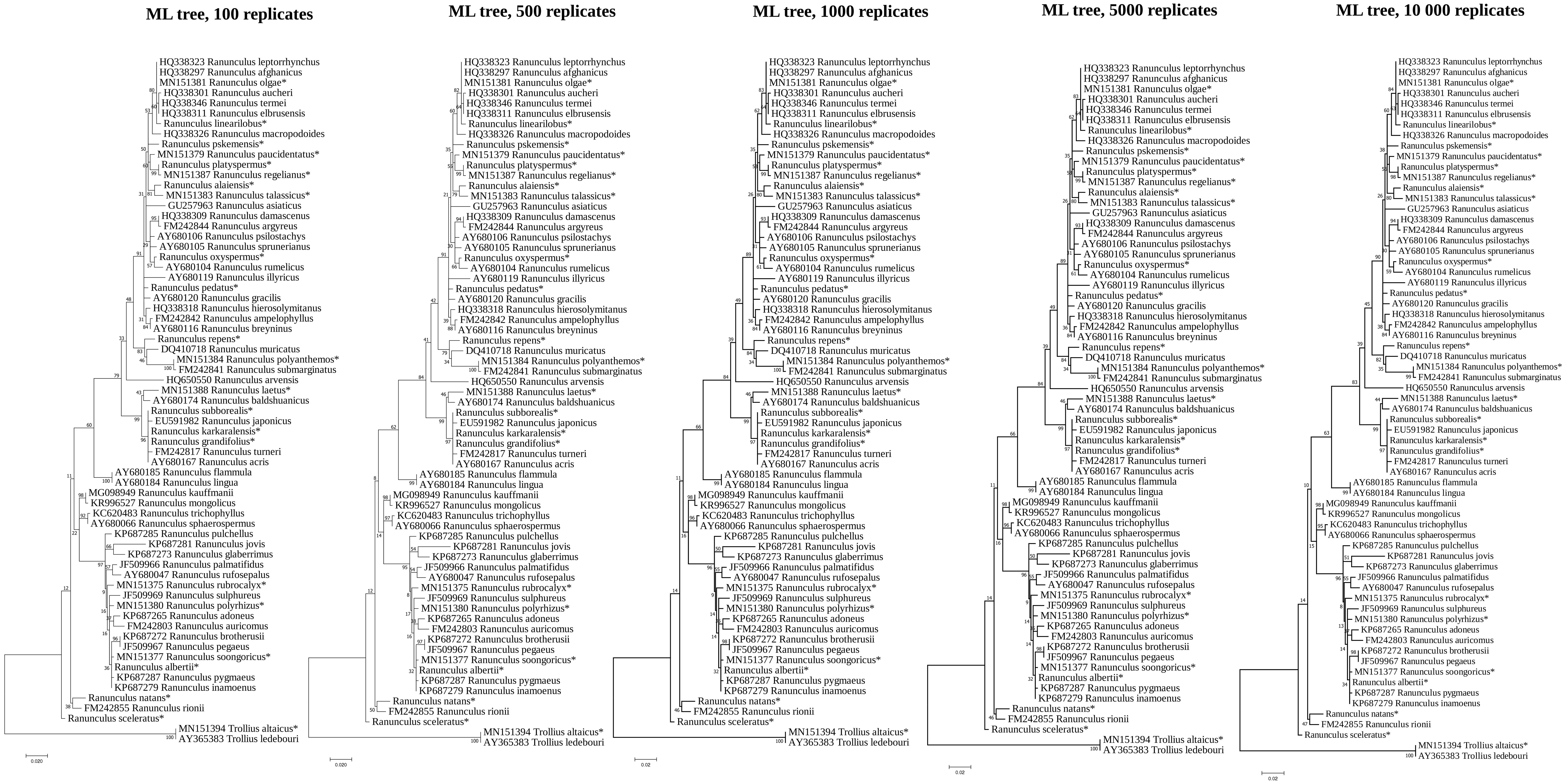
ML tree, 100 replicates
HQ338323 Ranunculus leptorrhynchus
HQ338297 Ranunculus afghanicus
MN151381 Ranunculus olgae*
HQ338301 Ranunculus aucheri
HQ338346 Ranunculus termei
HQ338311 Ranunculus elbrusensis
Ranunculus linearilobus*
HQ338326 Ranunculus macropodoides
Ranunculus pskemensis*
MN151379 Ranunculus paucidentatus*
Ranunculus platyspermus*
MN151387 Ranunculus regelianus*
Ranunculus alaiensis*
MN151383 Ranunculus talassicus*
GU257963 Ranunculus asiaticus
HQ338309 Ranunculus damascenus
FM242844 Ranunculus argyreus
AY680106 Ranunculus psilostachys
AY680105 Ranunculus sprunerianus
Ranunculus oxyspermus*
AY680104 Ranunculus rumelicus
AY680119 Ranunculus illyricus
Ranunculus pedatus*
AY680120 Ranunculus gracilis
HQ338318 Ranunculus hierosolymitanus
FM242842 Ranunculus ampelophyllus
AY680116 Ranunculus breyninus
Ranunculus repens*
DQ410718 Ranunculus muricatus
MN151384 Ranunculus polyanthemos*
FM242841 Ranunculus submarginatus
HQ650550 Ranunculus arvensis
MN151388 Ranunculus laetus*
AY680174 Ranunculus baldshuanicus
Ranunculus subborealis*
EU591982 Ranunculus japonicus
Ranunculus karkaralensis*
Ranunculus grandifolius*
FM242817 Ranunculus turneri
AY680167 Ranunculus acris
AY680185 Ranunculus flammula
AY680184 Ranunculus lingua
MG098949 Ranunculus kauffmanii
KR996527 Ranunculus mongolicus
KC620483 Ranunculus trichophyllus
AY680066 Ranunculus sphaerospermus
KP687285 Ranunculus pulchellus
KP687281 Ranunculus jovis
KP687273 Ranunculus glaberrimus
JF509966 Ranunculus palmatifidus
AY680047 Ranunculus rufosepalus
MN151375 Ranunculus rubrocalyx*
JF509969 Ranunculus sulphureus
MN151380 Ranunculus polyrhizus*
KP687265 Ranunculus adoneus
FM242803 Ranunculus auricomus
KP687272 Ranunculus brotherusii
JF509967 Ranunculus pegaeus
MN151377 Ranunculus soongoricus*
Ranunculus albertii*
KP687287 Ranunculus pygmaeus
KP687279 Ranunculus inamoenus
Ranunculus natans*
FM242855 Ranunculus rionii
Ranunculus sceleratus*
MN151394 Trollius altaicus*
AY365383 Trollius ledebouri
0.020
ML tree, 500 replicates
HQ338323 Ranunculus leptorrhynchus
HQ338297 Ranunculus afghanicus
MN151381 Ranunculus olgae*
HQ338301 Ranunculus aucheri
HQ338346 Ranunculus termei
HQ338311 Ranunculus elbrusensis
Ranunculus linearilobus*
HQ338326 Ranunculus macropodoides
Ranunculus pskemensis*
MN151379 Ranunculus paucidentatus*
Ranunculus platyspermus*
MN151387 Ranunculus regelianus*
Ranunculus alaiensis*
MN151383 Ranunculus talassicus*
GU257963 Ranunculus asiaticus
HQ338309 Ranunculus damascenus
FM242844 Ranunculus argyreus
AY680106 Ranunculus psilostachys
AY680105 Ranunculus sprunerianus
Ranunculus oxyspermus*
AY680104 Ranunculus rumelicus
AY680119 Ranunculus illyricus
Ranunculus pedatus*
AY680120 Ranunculus gracilis
HQ338318 Ranunculus hierosolymitanus
FM242842 Ranunculus ampelophyllus
AY680116 Ranunculus breyninus
Ranunculus repens*
DQ410718 Ranunculus muricatus
MN151384 Ranunculus polyanthemos*
FM242841 Ranunculus submarginatus
HQ650550 Ranunculus arvensis
MN151388 Ranunculus laetus*
AY680174 Ranunculus baldshuanicus
Ranunculus subborealis*
EU591982 Ranunculus japonicus
Ranunculus karkaralensis*
Ranunculus grandifolius*
FM242817 Ranunculus turneri
AY680167 Ranunculus acris
AY680185 Ranunculus flammula
AY680184 Ranunculus lingua
MG098949 Ranunculus kauffmanii
KR996527 Ranunculus mongolicus
KC620483 Ranunculus trichophyllus
AY680066 Ranunculus sphaerospermus
KP687285 Ranunculus pulchellus
KP687281 Ranunculus jovis
KP687273 Ranunculus glaberrimus
JF509966 Ranunculus palmatifidus
AY680047 Ranunculus rufosepalus
MN151375 Ranunculus rubrocalyx*
JF509969 Ranunculus sulphureus
MN151380 Ranunculus polyrhizus*
KP687265 Ranunculus adoneus
FM242803 Ranunculus auricomus
KP687272 Ranunculus brotherusii
JF509967 Ranunculus pegaeus
MN151377 Ranunculus soongoricus*
Ranunculus albertii*
KP687287 Ranunculus pygmaeus
KP687279 Ranunculus inamoenus
Ranunculus natans*
FM242855 Ranunculus rionii
Ranunculus sceleratus*
MN151394 Trollius altaicus*
AY365383 Trollius ledebouri
0.020
ML tree, 1000 replicates
HQ338323 Ranunculus leptorrhynchus
HQ338297 Ranunculus afghanicus
MN151381 Ranunculus olgae*
HQ338301 Ranunculus aucheri
HQ338346 Ranunculus termei
HQ338311 Ranunculus elbrusensis
Ranunculus linearilobus*
HQ338326 Ranunculus macropodoides
Ranunculus pskemensis*
MN151379 Ranunculus paucidentatus*
Ranunculus platyspermus*
MN151387 Ranunculus regelianus*
Ranunculus alaiensis*
MN151383 Ranunculus talassicus*
GU257963 Ranunculus asiaticus
HQ338309 Ranunculus damascenus
FM242844 Ranunculus argyreus
AY680106 Ranunculus psilostachys
AY680105 Ranunculus sprunerianus
Ranunculus oxyspermus*
AY680104 Ranunculus rumelicus
AY680119 Ranunculus illyricus
Ranunculus pedatus*
AY680120 Ranunculus gracilis
HQ338318 Ranunculus hierosolymitanus
FM242842 Ranunculus ampelophyllus
AY680116 Ranunculus breyninus
Ranunculus repens*
DQ410718 Ranunculus muricatus
MN151384 Ranunculus polyanthemos*
FM242841 Ranunculus submarginatus
HQ650550 Ranunculus arvensis
MN151388 Ranunculus laetus*
AY680174 Ranunculus baldshuanicus
Ranunculus subborealis*
EU591982 Ranunculus japonicus
Ranunculus karkaralensis*
Ranunculus grandifolius*
FM242817 Ranunculus turneri
AY680167 Ranunculus acris
AY680185 Ranunculus flammula
AY680184 Ranunculus lingua
MG098949 Ranunculus kauffmanii
KR996527 Ranunculus mongolicus
KC620483 Ranunculus trichophyllus
AY680066 Ranunculus sphaerospermus
KP687285 Ranunculus pulchellus
KP687281 Ranunculus jovis
KP687273 Ranunculus glaberrimus
JF509966 Ranunculus palmatifidus
AY680047 Ranunculus rufosepalus
MN151375 Ranunculus rubrocalyx*
JF509969 Ranunculus sulphureus
MN151380 Ranunculus polyrhizus*
KP687265 Ranunculus adoneus
FM242803 Ranunculus auricomus
KP687272 Ranunculus brotherusii
JF509967 Ranunculus pegaeus
MN151377 Ranunculus soongoricus*
Ranunculus albertii*
KP687287 Ranunculus pygmaeus
KP687279 Ranunculus inamoenus
Ranunculus natans*
FM242855 Ranunculus rionii
Ranunculus sceleratus*
MN151394 Trollius altaicus*
AY365383 Trollius ledebouri
0.02
ML tree, 5000 replicates
HQ338323 Ranunculus leptorrhynchus
HQ338297 Ranunculus afghanicus
MN151381 Ranunculus olgae*
HQ338301 Ranunculus aucheri
HQ338346 Ranunculus termei
HQ338311 Ranunculus elbrusensis
Ranunculus linearilobus*
HQ338326 Ranunculus macropodoides
Ranunculus pskemensis*
MN151379 Ranunculus paucidentatus*
Ranunculus platyspermus*
MN151387 Ranunculus regelianus*
Ranunculus alaiensis*
MN151383 Ranunculus talassicus*
GU257963 Ranunculus asiaticus
HQ338309 Ranunculus damascenus
FM242844 Ranunculus argyreus
AY680106 Ranunculus psilostachys
AY680105 Ranunculus sprunerianus
Ranunculus oxyspermus*
AY680104 Ranunculus rumelicus
AY680119 Ranunculus illyricus
Ranunculus pedatus*
AY680120 Ranunculus gracilis
HQ338318 Ranunculus hierosolymitanus
FM242842 Ranunculus ampelophyllus
AY680116 Ranunculus breyninus
Ranunculus repens*
DQ410718 Ranunculus muricatus
MN151384 Ranunculus polyanthemos*
FM242841 Ranunculus submarginatus
HQ650550 Ranunculus arvensis
MN151388 Ranunculus laetus*
AY680174 Ranunculus baldshuanicus
Ranunculus subborealis*
EU591982 Ranunculus japonicus
Ranunculus karkaralensis*
Ranunculus grandifolius*
FM242817 Ranunculus turneri
AY680167 Ranunculus acris
AY680185 Ranunculus flammula
AY680184 Ranunculus lingua
MG098949 Ranunculus kauffmanii
KR996527 Ranunculus mongolicus
KC620483 Ranunculus trichophyllus
AY680066 Ranunculus sphaerospermus
KP687285 Ranunculus pulchellus
KP687281 Ranunculus jovis
KP687273 Ranunculus glaberrimus
JF509966 Ranunculus palmatifidus
AY680047 Ranunculus rufosepalus
MN151375 Ranunculus rubrocalyx*
JF509969 Ranunculus sulphureus
MN151380 Ranunculus polyrhizus*
KP687265 Ranunculus adoneus
FM242803 Ranunculus auricomus
KP687272 Ranunculus brotherusii
JF509967 Ranunculus pegaeus
MN151377 Ranunculus soongoricus*
Ranunculus albertii*
KP687287 Ranunculus pygmaeus
KP687279 Ranunculus inamoenus
Ranunculus natans*
FM242855 Ranunculus rionii
Ranunculus sceleratus*
MN151394 Trollius altaicus*
AY365383 Trollius ledebouri
0.02
ML tree, 10 000 replicates
HQ338323 Ranunculus leptorrhynchus
HQ338297 Ranunculus afghanicus
MN151381 Ranunculus olgae*
HQ338301 Ranunculus aucheri
HQ338346 Ranunculus termei
HQ338311 Ranunculus elbrusensis
Ranunculus linearilobus*
HQ338326 Ranunculus macropodoides
Ranunculus pskemensis*
MN151379 Ranunculus paucidentatus*
Ranunculus platyspermus*
MN151387 Ranunculus regelianus*
Ranunculus alaiensis*
MN151383 Ranunculus talassicus*
GU257963 Ranunculus asiaticus
HQ338309 Ranunculus damascenus
FM242844 Ranunculus argyreus
AY680106 Ranunculus psilostachys
AY680105 Ranunculus sprunerianus
Ranunculus oxyspermus*
AY680104 Ranunculus rumelicus
AY680119 Ranunculus illyricus
Ranunculus pedatus*
AY680120 Ranunculus gracilis
HQ338318 Ranunculus hierosolymitanus
FM242842 Ranunculus ampelophyllus
AY680116 Ranunculus breyninus
Ranunculus repens*
DQ410718 Ranunculus muricatus
MN151384 Ranunculus polyanthemos*
FM242841 Ranunculus submarginatus
HQ650550 Ranunculus arvensis
MN151388 Ranunculus laetus*
AY680174 Ranunculus baldshuanicus
Ranunculus subborealis*
EU591982 Ranunculus japonicus
Ranunculus karkaralensis*
Ranunculus grandifolius*
FM242817 Ranunculus turneri
AY680167 Ranunculus acris
AY680185 Ranunculus flammula
AY680184 Ranunculus lingua
MG098949 Ranunculus kauffmanii
KR996527 Ranunculus mongolicus
KC620483 Ranunculus trichophyllus
AY680066 Ranunculus sphaerospermus
KP687285 Ranunculus pulchellus
KP687281 Ranunculus jovis
KP687273 Ranunculus glaberrimus
JF509966 Ranunculus palmatifidus
AY680047 Ranunculus rufosepalus
MN151375 Ranunculus rubrocalyx*
JF509969 Ranunculus sulphureus
MN151380 Ranunculus polyrhizus*
KP687265 Ranunculus adoneus
FM242803 Ranunculus auricomus
KP687272 Ranunculus brotherusii
JF509967 Ranunculus pegaeus
MN151377 Ranunculus soongoricus*
Ranunculus albertii*
KP687287 Ranunculus pygmaeus
KP687279 Ranunculus inamoenus
Ranunculus natans*
FM242855 Ranunculus rionii
Ranunculus sceleratus*
MN151394 Trollius altaicus*
AY365383 Trollius ledebouri
0.02

MP tree, 100 replicates

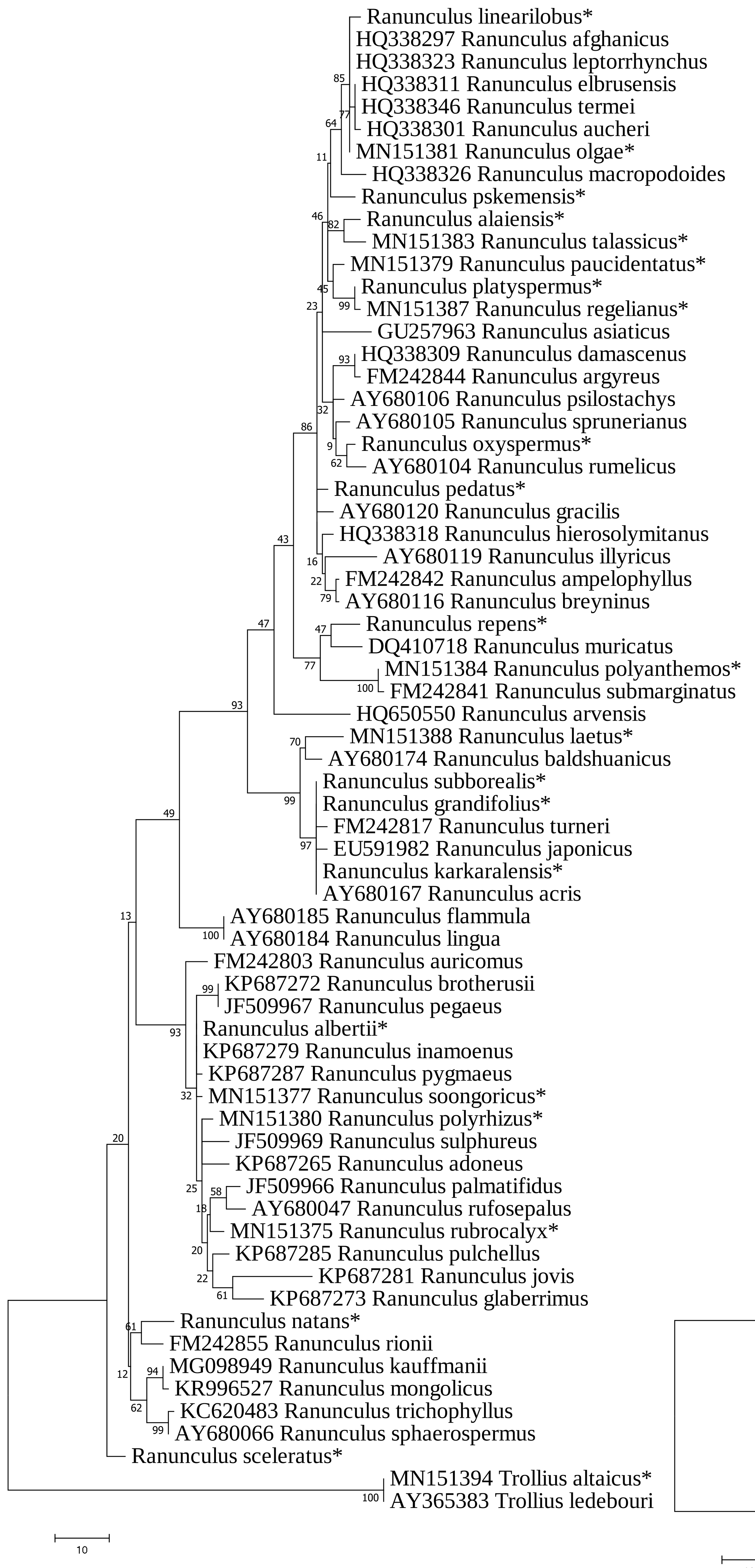

MP tree, 500 replicates

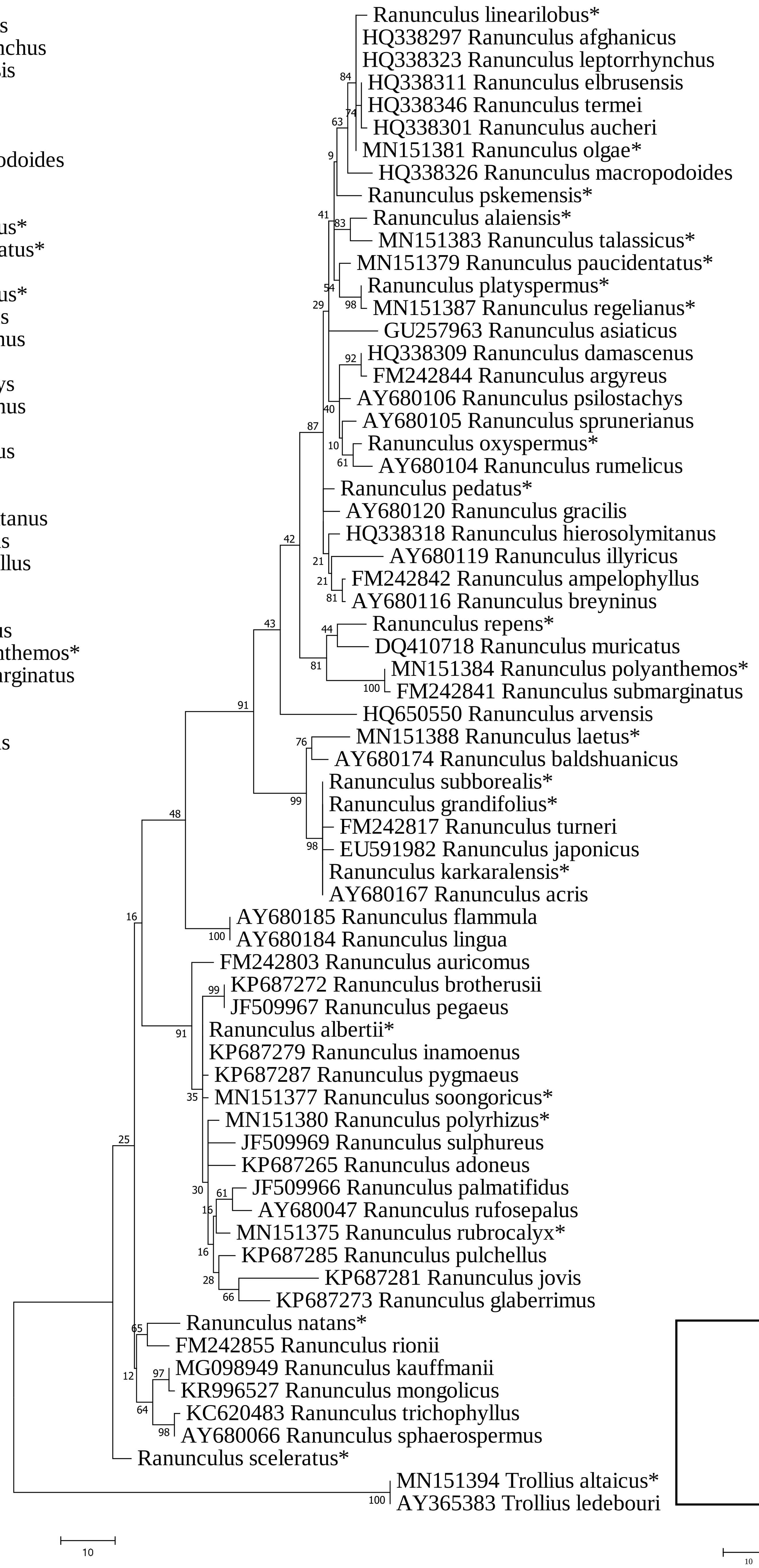

MP tree, 1000 replicates

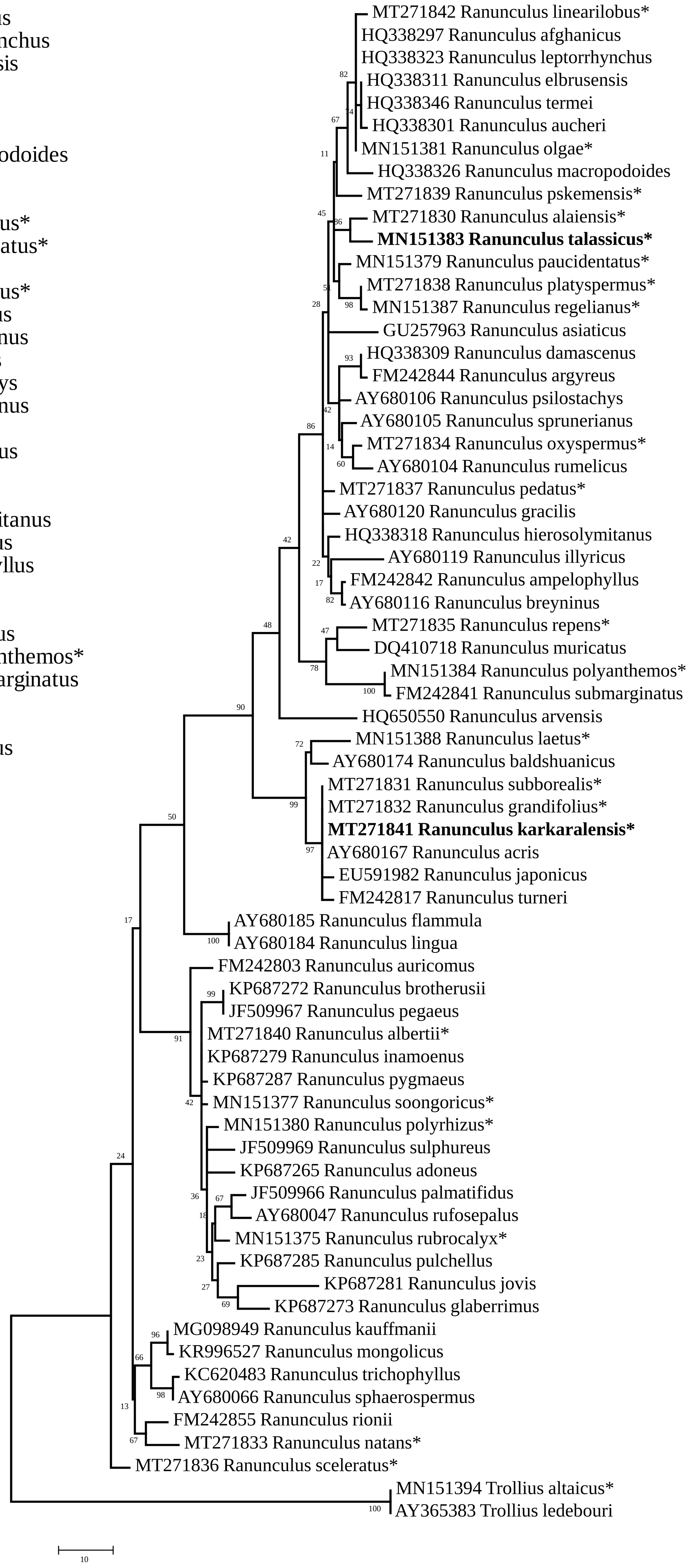

MP tree, 5000 replicates

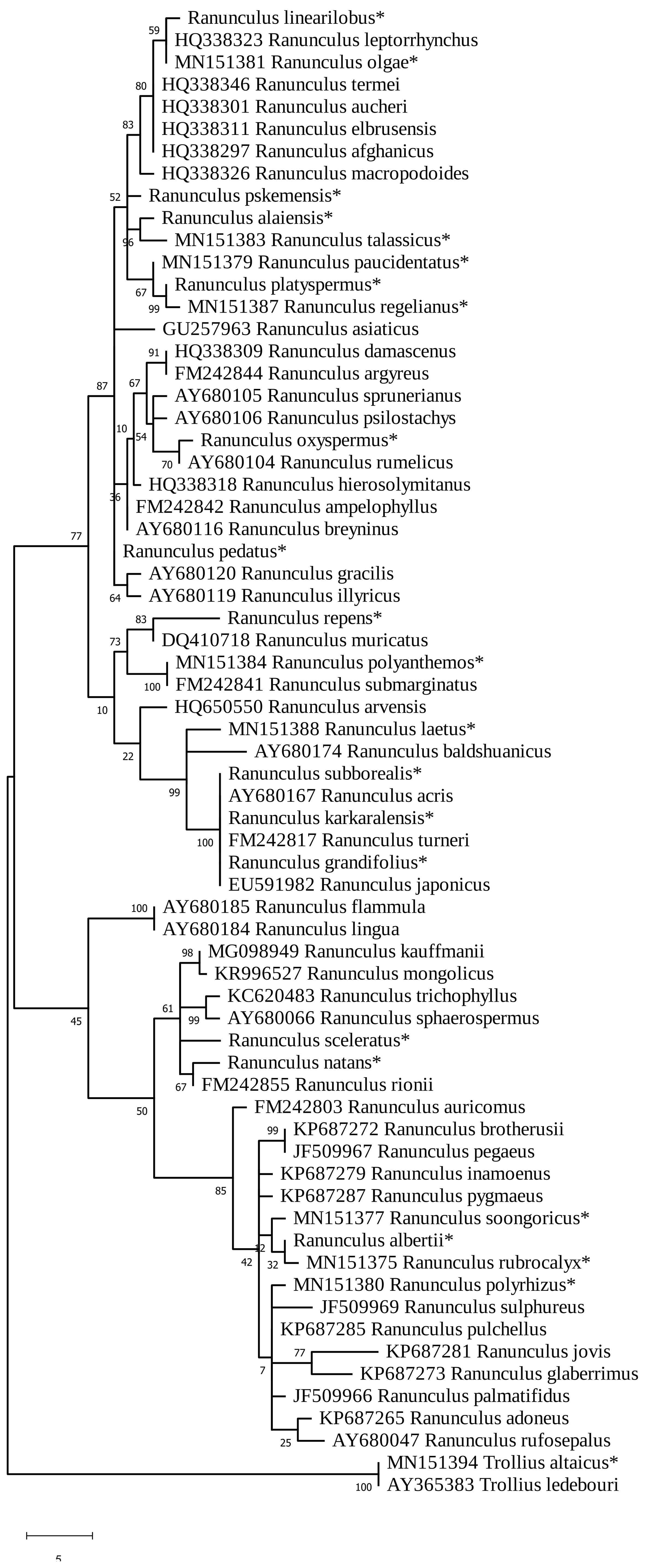

MP tree, 10 000 replicates

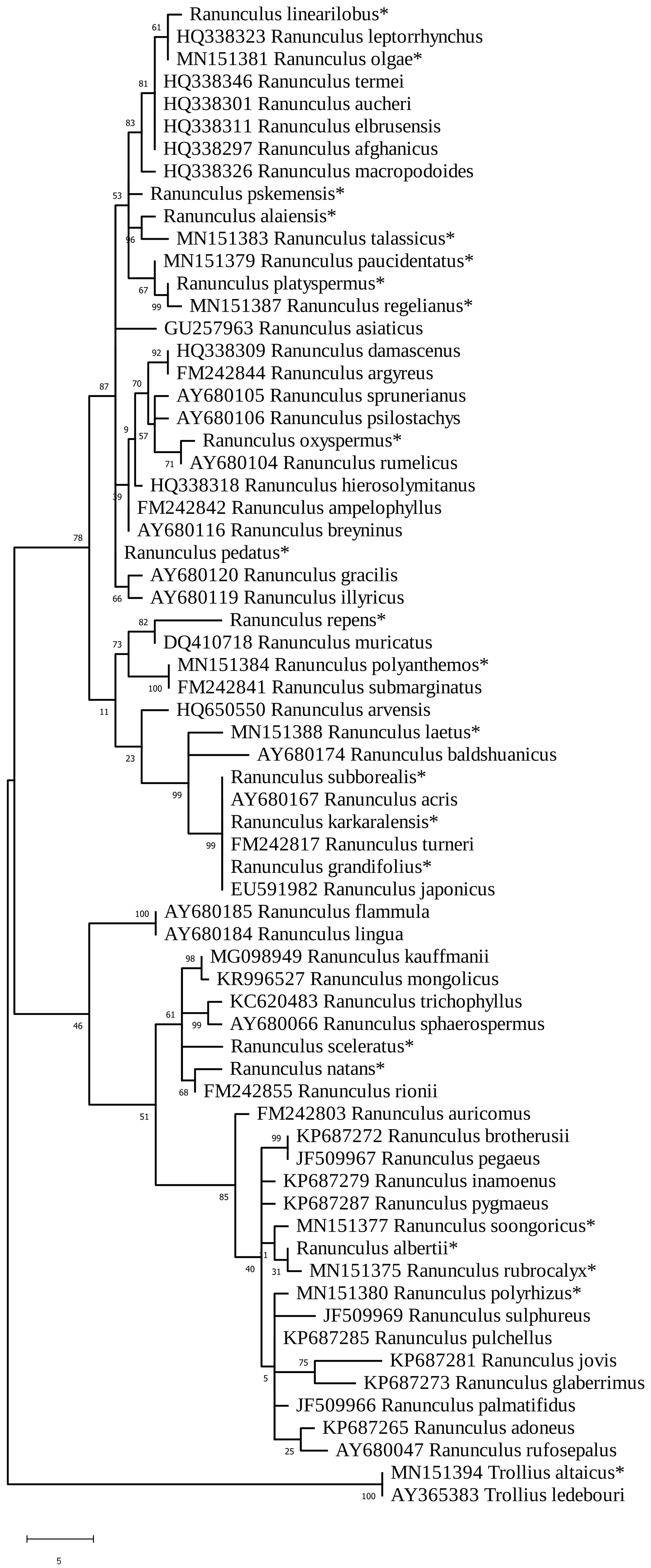

Supplement: S2 Appendix — (PDF) [file pone.0240121.s004.pdf]
